# *Musa* species in mainland Southeast Asia: From wild to domesticate

**DOI:** 10.1371/journal.pone.0307592

**Published:** 2024-10-02

**Authors:** Christophe Jenny, Gabriel Sachter-Smith, Catherine Breton, Ronan Rivallan, Jean-Pierre Jacquemoud-Collet, Cécile Dubois, Matthieu Chabannes, Ngọc-Sâm Lý, Thomas Haevermans, Tiến-Dũng Triệu, Oudomphone Insisiengmay, Ting Zhang, Marie-Line Caruana, Julie Sardos, Xavier Perrier

**Affiliations:** 1 CIRAD, UMR AGAP Institut, Montpellier, France; 2 UMR AGAP Institut, Univ Montpellier, CIRAD, INRAE, Institut Agro, Montpellier, France; 3 Hawaii Banana Source, Ohau, Hawai`i, United States of America; 4 Bioversity International, Parc Scientifique Agropolis II, Montpellier, France; 5 Department of Biological Resources, Institute of Tropical Biology, Vietnam Academy of Science and Technology, Hồ Chí Minh City, Vietnam; 6 Institut de Systématique Évolution Biodiversité (ISYEB), Muséum National d’histoire Naturelle, Centre national de la Recherche Scientifique, École Pratique des Hautes Études, Université des Antilles, Sorbonne Université, Paris, France; 7 Northern Mountainous Agriculture and Forestry Science Institute, Phú Hộ, Vietnam; 8 The Cabinet of the Lao Academy of Science and Technology, Ministry of Education and Sport, Vientiane Capital, Lao PDR; 9 Germplasm Bank of Wild Species, Kunming Institute of Botany, Chinese Academy of Sciences, Kunming, China; 10 CIRAD, Montpellier, France; ICAR - Central Tobacco Research Institute, INDIA

## Abstract

Many species are defined in the *Musa* section within its natural diversification area in Southeast Asia. However, their actual number remains debated as botanical characterisation, distribution and intraspecific variability are still poorly known, compromising their preservation and their exploitation as crop wild relatives of cultivated forms. To address the underexplored *Musa* diversity in mainland Southeast Asia, at the northern edge of the natural range, 208 specimens were collected in Vietnam, Laos and China, mainly belonging to *Musa balbisiana*, *M*. *itinerans*, *M*. *acuminata* and *M*. *yunnanensis*. Data on location, morphology, environment and local knowledge were recorded, and leaf samples collected for high-throughput genotyping. This study combines geographical, morphological, and genomic diversity to clarify the taxonomic classification. The collected species exhibit highly distinctive morphologies and genomes, just as they differ in ranges and life traits. Intraspecific genomic diversity was also observed, although not necessarily morphologically perceptible. Mainland Southeast Asia is confirmed as a primary diversification centre for the *Musa* section. The diversity observed is only partially represented in major international *ex situ* collections, calling for their urgent enrichment and the promotion of *in situ* management procedures, for the protection of these threatened species and to better harness their potential in breeding programmes. Although considered wild, the species studied are all affected to varying extents by human use. *Musa yunnanensis* and *M*. *acuminata* subsp. *burmannica* are the most strictly wild forms, with spontaneous interspecific hybrids first described in this study. Although gathered as fodder, they were only occasionally dispersed outside their endemic zones. *Musa itinerans* is not cultivated *per se*, but natural populations are widely exploited, leading to a geographically structured diversity. The diversity of *M*. *balbisiana* is widely distributed and geographically structured by human activities. This species should be regarded as domesticated. These various stages, from simple opportunistic gathering to true domestication, shed light on the evolutionary history of today’s cultivated varieties.

## Introduction

Plants in the family *Musaceae* Juss. are giant herbaceous monocots. Three genera are recognized in this family: *Ensete* (Bruce) Horan, *Musella* Franch., and *Musa* L. Botanists have described several species in all three genera based on morphological characteristics. The seven *Ensete* species are found in Asia and tropical Africa, notably in Ethiopia where *Ensete* forms are traditionally cultivated for their corms as a starch-rich food source [1]. The genus *Musella*, limited to only one species *Musella lasiocarpa* Franch., is present in southern China [2] where it is cultivated for its ornamental flower; it is sometimes considered monophyletic with *Ensete* [3]. The genus *Musa*, the most diverse with more than 70 species, is native to the Asian continent, primarily mainland Southeast Asia (mSEA) and island Southeast Asia (iSEA), extending to the eastern and southern Indian subcontinent and to Near Oceania, as far as the Solomon Islands. Within the genus *Musa*, section *Callimusa*, which combines the former 9- and 10-chromosome sections *Callimusa* and *Australimusa* [4] is distributed in iSEA and Oceania. Section *Musa*, which combines the former 11-chromosome sections *Eumusa* and *Rhodochlamys* [4], is largely distributed from Northeast India to Near Oceania.

Although there is now a consensus on the major species, debate continues among specialists, with new species regularly being proposed and others synonymised [5–7]. The recent publication of a comprehensive review of the literature [8], leads to the conclusion that further *in situ* observations are still needed to fully resolve the taxonomic organisation of the genus.

The use of molecular markers has largely confirmed and occasionally refined the taxonomy of *Musaceae*. More recently, new genomic resources have been released. The first assembled genome was a doubled haploid of ‘Pahang’, a *M*. *acuminata* subsp. *malaccensis* (Ridl.) N.W. Simmonds [9], later updated [10]. Recently, using long read sequencing, the genomes of each haplotype of a *M*. *acuminata* subsp. *malaccensis* were published [11], accounting for the heterozygosity of the species. Fully assembled genomes were also published for *M*. *balbisiana* Colla, *M*. *itinerans* Cheesman, *M*. *beccarii* N.W. Simmonds, as well as draft assemblies for *M*. *schizocarpa* N.W. Simmonds, *M*. *textilis* Née, and *E*. *glaucum* (Roxb.) Cheesman in the genus *Ensete* (see [12] for an updated review). These genomes are available on “The Banana Genome Hub” [13, 14]. These assembled reference genomes provide access to various structural markers of diversity, such as specific chromosomal rearrangements (translocations, inversions) [15]. The complete sequences also enabled locating numerous genome-wide single nucleotide polymorphisms (SNPs), allowing genotyping-by-sequencing (GBS) methods to be easily applied to the large sample sizes required for diversity studies. Chloroplast genomes have also been sequenced, providing fully resolved phylogenetic trees, based on the maternal lineage, for the *Musaceae* family [12].

Most of these studies rely on the same set of accessions available in most *ex situ* collections. Consequently, they ignore a significant part of the plant diversity in natural habitats [16], specifically in geographically or politically challenging areas. Additionally, these studies are sometimes confused by the approximate identification of these accessions within a taxonomic system that is not yet clearly established, as observed in some critical genera groups [17–19].

Within section *Musa*, the focus of this study, botanists have described, based on morphological characters and genomic evidence, a series of species spontaneously present across the entire range of the family *Musaceae*. Among the genus *Musa*, *Musa acuminata* and *M*. *balbisiana* are the best characterized, having long been recognized as the two main progenitors of almost all major groups of banana cultivars grown today [20, 21].

Bananas are a key crop in all humid subtropical regions, with around 140 million tonnes produced globally [22]. They are cultivated partly for the international sweet banana market but mainly for individual consumption and local markets, providing a staple food for smallholders across vast areas [23]. Banana varieties are cultivated for their edible fruits, with domestication syndrome combining sterility (or at least extremely low fertility) and parthenocarpy, balanced by the active vegetative propagation present in most *Musa* species [24].

*Musa acuminata* (A genome) is found, alone or with other *Musa* genomes, in all edible cultivars. Several subspecies are often defined [25–27], reflecting differentiation by geographic isolation as the species spread from north-western mainland Southeast Asia across the highly insular Southeast Asia [28–30]. Diploid cultivars derived from *M*. *acuminata* (AA cultivars) are assumed to be the most primitive cultivated forms. They are intersubspecific hybrids [15, 31, 32], resulting from crosses between *acuminata* subspecies naturally isolated but brought into contact as human populations migrated across the region, as traceable through linguistics [33]. Among these subspecies, three—*banksii*, *zebrina* and *malaccensis—*are considered the main contributors, though other subspecies may have contributed to particular cultivars. Moreover, at least two other contributing gene pools have recently been suggested [15, 32]. A third uncharacterized contributing pool around North Borneo has also been proposed [30, 34].

Secondary hybridisation with *M*. *balbisiana* (B genome) also occurred locally, producing AB diploid cultivars. Occasional contributions from other species have also been observed, such as *M*. *schizocarpa* introgressions deriving from its initial spontaneous hybridisation with *M*. *acuminata* subsp. *banksii* (F. Muell.) N.W. Simmonds, sympatric in Papua New-Guinea (PNG) [31, 35].

The association of distant genomes in these inter(sub)specific diploid hybrids induces distorted meiosis, contributing to sterility and the formation of unreduced gametes [36], leading to the formation of triploid cultivars [37, 38]. These triploid cultivars are the most widely cultivated forms today. They form different clonal groups [39] which are only somaclonally diversified, showing changes in phenotypic expression that are stable through vegetative propagation.

To address the multiple biotic and abiotic constraints currently endangering banana production in many areas, breeding programmes were initiated several years ago [24]. However, the mandatory sterility of cultivated varieties requires relying on fertile wild relatives to restore cross-breeding capacity. It is therefore essential to select the most suitable wild forms according to breeding objectives. This requires a thorough knowledge of ancestral forms involved in the domestication process, especially as wild contributors appear to be more diverse than previously thought and remain incompletely identified.

However, the geographical ranges of *Musa* species are not always known precisely, and moreover, the intraspecific variability in their respective distribution areas is often poorly documented. This confusion compromises both *in situ* and *ex situ* conservation of these species and their use as crop wild relatives in breeding programmes.

Within the broad range of the genus *Musa*, Papua New Guinea has been the subject of several extensive collecting missions and studies [40–42], as have The Philippines [43], Thailand [44–46], and China [47, 48].

The present study focuses on the northern geographical part of the *Musa* range, at the junction of Vietnam, Laos, and China’s Yunnan province. This area has been only partially explored yet is particularly rich in *Musa* species as the cradle of the genus. Additionally, the region is seriously affected by deforestation and biodiversity loss due to the destruction of natural habitats.

Two collecting missions were conducted in partnership with the countries concerned: the first in Vietnam and the second in Laos and China. These missions aimed to sample all *Musa* species found at the many sites visited in each country. Emphasis was placed on *M*. *balbisiana* whose known diversity is extremely limited despite its involvement in many cultivated hybrids. Several other native *Musa* species were also collected: *M*. *itinerans*, *M*. *acuminata*, *M*. *yunnanensis* Häkkinen & Wang Hong, *M*. *rubra* Kurz and some other species of the former *Rhodochlamys* section.

Geographical location, morphological characteristics, environmental conditions, and local knowledge, when available were recorded as described below. High-density genotyping was performed on all collected specimens. This approach, combining morphological and genomic diversity on large populations while accounting for intraspecific variability, is quite innovative in *Musa*. It therefore provides a solid basis for an in-depth understanding of the concerned species and subsequent taxonomic classification. The results obtained are essential for future *ex situ* and *in situ* biodiversity conservation projects and open possibilities for better utilisation of crop wild relatives in breeding programmes [49, 50]. They also illustrate the early stages of domestication processes in the *Musaceae* family, from simple opportunistic gathering to true domestication.

## Material and methods

### Collecting missions

**Fig 1:**
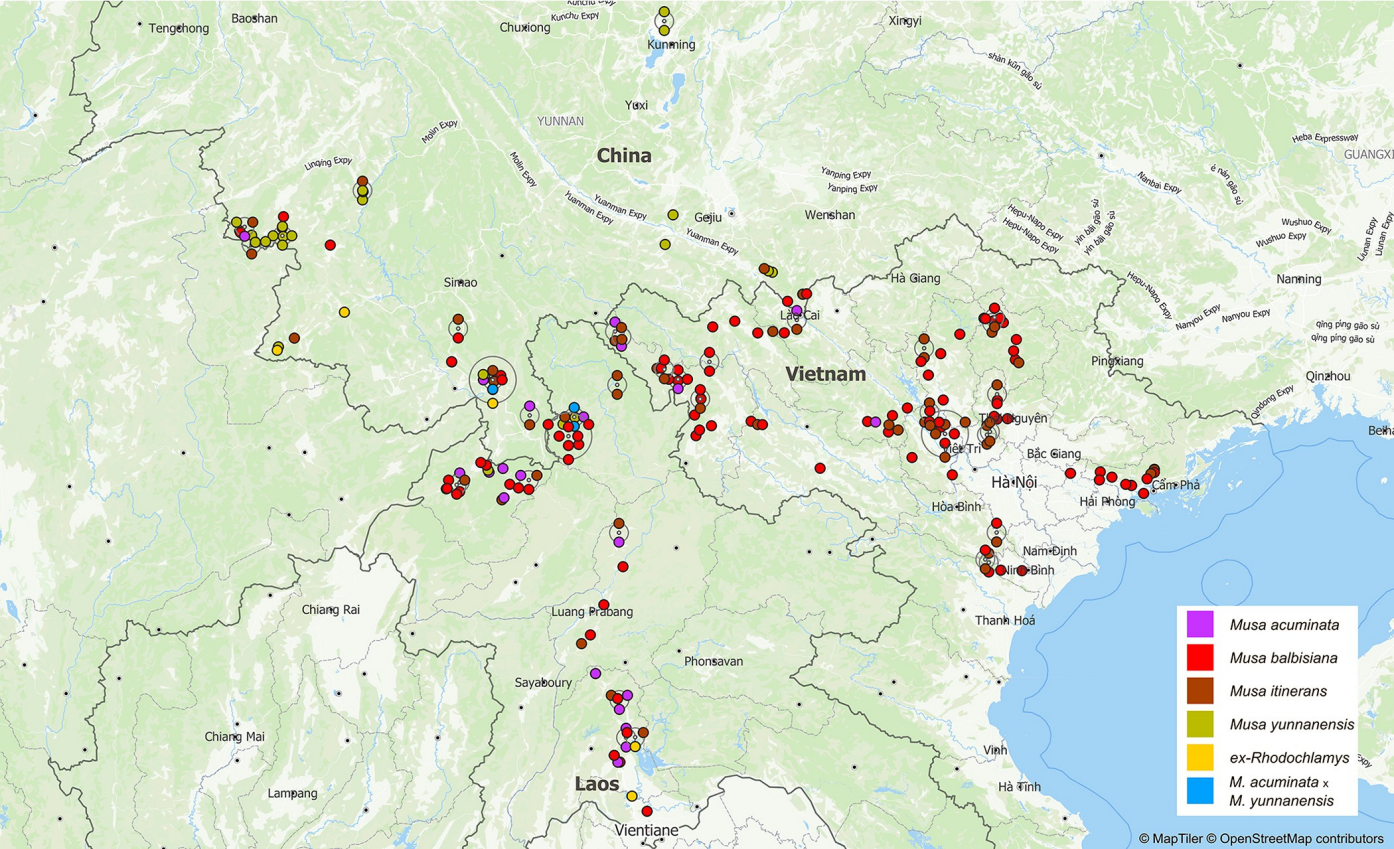
Geographical distribution of the 220 studied specimens. Colours by species, according to the legend. Shifting of overlapping points using QGIS internal displacement option. Terrain data sourced from OpenStreetMap. Map projection: WGS 84.

The collecting missions were conducted in two phases. The first phase, in 2018, took place in North Vietnam and was the most significant in terms of collected material. It spanned over 400 km from Ha Long Bay in the East to the borders with Laos and China in the Northwest (**Fig 1**). The second phase, in 2019, occurred in Laos, extending from Vientiane up north to the borders of Vietnam and Myanmar, and then into China for exploration in Yunnan Province (**Fig 1**).

The collecting missions were carried out by travelling along roads and collecting all *Musa* material of interest as identified by the team or indicated by local farmers. This material was found in forest, along roadsides, in farmers’ fields or in gardens near houses. For each site, the team proposed a taxonomic classification, recorded the geolocation and administrative reference, and noted the morphological characteristics and context of discovery. Additionally, the team gathered available elements of local knowledge, such as vernacular names, uses, and origins, and took photographs of the environment, the whole plant and its characteristic details. A portion of the leaf blade (about 10 cm^2^) was placed in a paper bag, dried in silica gel for 48 hours, and then kept in dry conditions until it was stored at -80°C for genomic analysis. When available and with the agreement of the farmers, a plant sucker was also collected. These suckers were replanted for preservation in existing *ex situ* collections in Vietnam (Nomafsi Institute) and China (Kunming Institute of Botany), and a new field collection was created for this purpose at the Lao Academy of Science and Technology in Vientiane.

Although the three countries surveyed have ratified the Nagoya Protocol, the rules on access to genetic resources and associated traditional knowledge are specific to each country. The necessary procedures were completed in advance in each case, authorizing the collection of dried leaf samples, their transfer to France for genomic analysis, and their study by the various project partners.

For the collecting mission in Vietnam, an application for access to genetic resources was signed in 2018 between one partner from Vietnam (the Institute of Tropical Biology–ITB), the French Agricultural Research Centre for International Development (CIRAD), and the National Museum of Natural History of Paris (MNHN). This document was signed by the three directors of these scientific institutions. ITB was responsible for establishing contracts with the parks and reserves where applicants collected banana samples. CIRAD was responsible for the molecular characterisation of the banana samples, and MNHN was responsible for the long-term storage of the banana samples. Additionally, a collecting permit (No. 1961/TCLN-DDPH) was issued by the Vietnamese Ministry of Agriculture and Rural Development on 9 November 2018.

For the collecting mission in Laos, an application for access to genetic resources was signed in 2018 between the Institute of Tropical Biology (ITB) for Vietnam, the French Agricultural Research Centre for International Development (CIRAD), the National Museum of Natural History of Paris (MNHN), and the Cabinet of the Lao Academy of Science and Technology (CLAST). CLAST is a public establishment responsible for national scientific research projects and the promotion of cultural and scientific activities. Therefore, it was responsible for establishing contracts with the parks and reserves where applicants collected banana samples. The application for access to genetic resources was signed by the four directors of these scientific institutions. Additionally, a Material Transfer Agreement was also signed between the directors of CLAST and CIRAD in March 2019.

For the collecting mission in China, a Material Transfer Agreement was signed in 2019 between the directors of the Kunming Institute of Botany (KIB), CIRAD, and MNHN. Finally, a consortium agreement for the implementation of each of the two research projects (the Sud Expert Plantes Développement Durable programme through the DivBa SEP2D project (AAP3-97) and the BforBB open science project supported by the Agropolis Fondation under the reference ID 1605–011 through the “Investissements d’avenir” programme (Labex Agro:ANR-10-LABX-0001-01)) that financed the research described in our article was signed by ITB, MNHN, CIRAD, and the Northern Mountainous Agriculture and Forestry Science Institute (NOMAFSI) from Vietnam.

### Data organization

A significant amount of data was generated from field collections, genotyping processes and bioinformatics and statistical analysis procedures, as described below. A specific interactive mapping tool was developed in R-Shiny to represent the geographically structured data. This tool allows for interactive multi-criteria selection of the samples to be displayed and their graphical illustration using sets of colours to depict the modalities of several variables of interest (geographical information, taxonomic group, and genomic group). The geographical representations shown in this article were refined using QGIS 3.28.0 with the Outdoor layer provided by MapTiler & OpenStreetMap. The QGIS internal displacement option was used to display overlapping points.

### Genotyping by sequencing

The genomic characterisation of the samples was performed using a high-throughput genotyping method, specifically resequencing targeted portions of the genome through the GBS (Genotyping By Sequencing) technique [51].

DNA from each sample was extracted following a modified CTAB protocol [52]. Genotyping was conducted according to published procedures [51]. The GBS libraries based on StdGBS methods were constructed following standard protocols with the PstI and MseI restriction enzymes. The 300–500 bp short-insert libraries were sequenced in 150 bp paired-end reads using Illumina HiSeq2500 (Illumina, San Diego, CA, USA) by Genewiz, Azenta Life Sciences, USA.

The first GBS analysis (batch 1) was performed on the 182 samples collected in Vietnam in 2018. A set of 34 references representative of the genetic diversity in the genus *Musa* was added to insert the observed diversity within the global diversity. A second GBS analysis (batch 2) was carried out on the 153 samples collected during the 2019 survey in Laos and China. This analysis included 32 references (27 of which were identical to batch 1) and seven samples from batch 1, whose DNA quality had been insufficient in the previous analysis. In each batch, all samples were analysed twice to increase the sequencing depth, except for some genetically more distant samples (*Ensete*, *Callimusa and Musa* from the former *Rhodochlamys* section). Five samples were repeated on each plate to serve as internal repeatability controls.

### Bioinformatics procedures

After demultiplexing with GBSX [53], each of the ’paired end’ sequencing files (fastq_R1 and fastq_R2) was checked using FASTQC software [54] available at https://www.bioinformatics.babraham.ac.uk/projects/fastqc, for quality metrics, presence of adapters, and GC content. The files were then cleaned to remove Illumina adapter sequences and low-quality ends (Phred score > 20) using Cutadapt [55]. Reads shorter than 30 bp after trimming were discarded. The cleaned reads were aligned against the v2 genome of Pahang-HD, a doubled haploid from *M*. *acuminata malaccensis ‘*Pahang’ [9] obtained from the Banana Genome Hub [13, 14] using BWA-MEM [56]. Re-alignment was performed using the IndelRealigner module from GATK v4.1 [57]. The recommended GATK pipeline for non-model organisms was followed, including a recalibration step. This involved an initial round of SNP calling on the original uncalibrated data, selecting the SNPs with the highest confidence, and then executing a round of base recalibration on the original mapped read files. For duplicate specimens, a script using Sambamba software was employed to merge the recalibrated bam alignment files. SNP and indel calling were performed using the GATK module HaplotypeCaller v4.1. Subsequently, a script (gVCF2vcf -gz.pl) was written to combine the individual gVCF files into a single VCF file. The GenomicDB procedure from GATK was used to build the gVCF SNP database and the GenotypeGVCF procedure was then used to extract only the variant positions. The snpcluster exclusion procedure was applied to process SNP clusters, set with a threshold of three or more SNPs per 10 bp window. These procedures have been compiled in the pipeline Musa_NGS_Suite available at https://github.com/CathyBreton/Genomic_Evolution [58].

### Data analysis

The Musa_NGS_Suite was utilised to process the Vietnam and Laos/China specimen datasets separately, as they were obtained from two distinct analytical batches, producing two raw VCF files.

Using the GBS-Div software (for a detailed description, see **S1 File**), a filtered VCF file was extracted for each batch, retaining only bi-allelic sites with depths between 20 and 300. For any given site, only alleles representing at least 5% of the total depth at the site were kept. The two files were subsequently merged into a final VCF file keeping only the SNPs found to be polymorphic in both batches simultaneously.

Data analysis of the merged VCF file was also performed using GBS-Div. Statistical indicators for missing data, allele frequencies, depth distributions and ploidy ratios were applied to clean and validate the data. The ploidy ratios were also used to confirm the diploid status of all specimens. A dissimilarity matrix was calculated between pairs of specimens as the ratio of the number of identical alleles (0, 1 or 2) at a SNP to the total number of SNPs present for both specimens. A principal coordinate analysis (PCoA) was conducted on the dissimilarity matrix to provide an overall representation of the collected diversity. A Neighbour-Joining tree [59] was inferred to describe the relationships at the specimen level. This method was chosen because it is free of the assumptions usually required about the underlying evolutionary process, which are often unmet, or only partially met in plants that propagate clonally by budding and whose seeds and suckers may have been selectively disseminated by humans. An a posteriori least-squares re-estimation of edge length was then performed. The high number of markers limiting the efficiency of the bootstrap method, instead, other indicators were used to assess the reliability of the inferred tree. First, a Jackknife resampling procedure was applied to the specimens to detect influential units, measured by the impact of their removal on the tree’s topology. The tree model, which assumes no exchanges between different branches, was a priori considered valid since applied here at the species level. However, reticulations between tree nodes that significantly improved the fit to observed dissimilarities were sought as indicators of potential horizontal exchanges. All analyses were performed using DARwin software [60].

A standard genomic profile for the main pools (species or subspecies) was constructed as the majority consensus of the specimens considered reliable representatives of the pool.

Diagnostic SNPs that discriminate these pools were then determined as the loci that were homozygous for one allele in the pool consensus and homozygous for the alternate allele in all other pool consensuses. Finally, the genotypes at these diagnostic positions were used for chromosome painting of all specimens not involved in the construction of pool consensuses, to detect potential contributions of these major pools to those unassigned specimens. Both DARwin and GBS-Div software are freely available at: https://darwin.cirad.fr/.

## Results and discussion

A subset of 220 of *Musa* species representatives was selected for this study, from the 334 specimens collected which also included representatives of other genera within the family, those from the *Callimusa* section as well as cultivated bananas (**Fig 1** and **Table 1** for their geographical distribution in the study area).

**Table 1:** Summary of collected specimens and genotyped specimens/external references (ext ref), grouped according to former *Musa* sections.

*Musa* taxa	collected				GBS genotyped	
	Vietnam	Laos	China	Total	collected	ext ref
*M*. *balbisiana*	68	26	12	106	101	10
*M*. *itinerans*	35	11	10	56	41	1
*M*. *acuminata burmannica*	6	13	3	22	19	4
other *M*. *acuminata* subspecies						14
*M*. *yunnanensis*		3	22	25	22	
*M*. *acuminata × yunnanensis*		2	1	3	3	
*M*. *rubra/laterita*		1		1	1	1
*M*. *ornata*		2		2	1	1
*M*. *rubinea/sanguinea*			4	4	4	1
*M*. *velutina*			1	1	1	

Among the 21 genotyped external references, 11 were analysed using the same DNA in each of the two batches. These two repeats were not merged and are therefore present in duplicate in the dataset to check the repeatability of the genotyping process. Consequently, the final dataset contained 32 external references in addition to the 220 collected specimens (**S1 Table**).

The genotypes of 208 of these specimens (12 had insufficient amounts of extracted DNA) and the 32 external references were extracted from the global VCF file. The resulting VCF file on these 240 individuals is available on the Gigwa application [61].

On average, 156,418 valid SNPs were observed per sample. Based on the distribution of these values, a minimum threshold of 30,000 SNPs was set to retain a sample. This rule excluded 15 individuals from the analysis (mainly *M*. *itinerans* samples from the Vietnam batch). The depths at each locus ranged from 32 to 78, with an average depth of 48.3. These high depths were achieved by merging the two sequencing replicates of the same sample, the lowest depths corresponded to the few individuals analysed only once. These depths allowed for reliable estimates of ploidies and genotypes. The numbers of valid SNPs, depths by SNP, and heterozygosities (number of biallelic SNPs over the number of valid SNPs) for the 225 eligible individuals are given in **S1 Table**.

The Pahang-HD reference used for mapping may potentially introduce a bias for species other than *M*. *acuminata*. The average number of valid SNPs was logically highest for *M*. *acuminata*, around 180.000, but was equally high for *M*. *balbisiana*, likely due to the large number of specimens in the study. It was still close to 120,000 for *M*. *itinerans* and *M*. *yunnanensis* (**Table 2**), with similar sequencing depths across all the species. The numbers of shared SNPs are sufficient to perform reliable diversity analyses, making the Pahang HD reference an effective common reference for species whose genomic proximity is still high.

**Table 2:** Genotype comparison by species: Mean, min and max for valid SNP numbers, depth of sequencing, and heterozygosity (H%).

*Musa* taxa	n	valid SNPs			depth by SNP			H%		
		mean	min	max	mean	min	max	mean	min	max
*M*. *acuminata*	37	178 316	73 561	255 201	49.2	34.5	78.1	3.02%	0.50%	8.24%
*M*. *balbisiana*	121	179 440	90 246	230 406	49.6	37.0	57.6	3.26%	1.29%	5.25%
*M*. *itinerans*	42	117 599	39 063	205 154	43.7	32.2	58.3	2.58%	0.88%	3.75%
*M*. *yunnanensis*	22	116 424	41 286	190 133	45.2	32.9	59.1	1.54%	0.80%	2.58%
*Rhodochlamys sp*.	8	102 026	60 292	139 157	43.3	36.2	50.0	1.60%	0.28%	2.56%

**Fig 2:**
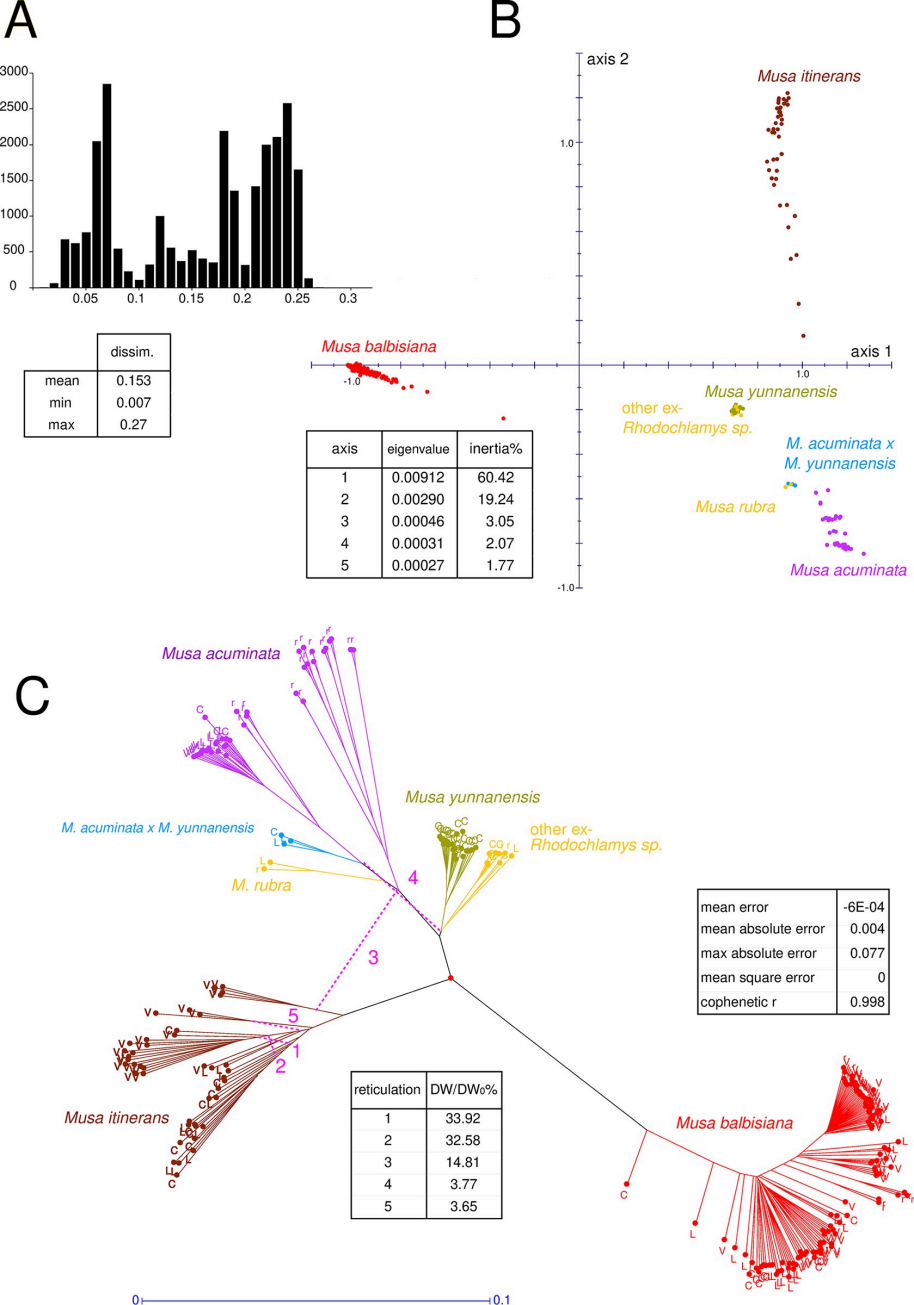
Dissimilarities between the 225 validated genotypes. (A) Dissimilarity distribution and parameters (B) Principal Coordinates analysis (PCoA), sample representation on axes 1 and 2. (C) Unrooted NJ Tree enhanced with the 5 best reticulations. Samples identified according to their origin: C: China, L: Laos, V: Vietnam, r: reference.

A two-by-two dissimilarity matrix was calculated for all pairs of the 225 validated individuals. On average over all pairs of individuals, 106,000 loci were available for the dissimilarity measure, with almost 90% of the pairs having between 80.000 and 170,000 SNPs in common. The distribution of these dissimilarities was multimodal, with a primary peak for intraspecific pairs and two peaks for interspecific pairs, the highest corresponding to pairs involving a *M*. *balbisiana* specimen (**Fig 2A**). The first PCoA plane (axes 1 and 2), accounting for almost 80% of the total variation, clearly separated the different species. Axis 3 (not shown) distinguished within *M*. *acuminata*, the subspecies *burmannica* N.W. Simmonds from the other subspecies (**Fig 2B**). The unrooted NJ tree was highly structured with well-differentiated species (**Fig 2C**). The high cophenetic correlation indicated that the dissimilarities were well adjusted by the tree distances. The procedure for detecting influential points highlighted only *M*. *acuminata* subsp. *truncata*, with a local impact on the cluster of *M*. *acuminata* subspecies other than *burmannica*. Several notable reticulations were detected, showing local deviations from the strictly dichotomous tree model. The most significant reticulations 1 and 2, which reduced the quadratic error for the concerned units by more than 30%, were internal to the Itinerans group, indicating that the Itinerans clusters in the tree are not so strictly divergent. The third reticulation (15%) indicated that a subset of *M*. *itinerans* was more closely related to the *M*. *acuminata* pole than the tree suggested. The fourth iteration (4%) logically strengthened the relationship between the hybrids *M*. *acuminata* x *M*. *yunnanensis* and the parent *M*. *yunnanensis*. The last reticulation (3%) was also internal to the Itinerans cluster.

Given the strong structure shown by the NJ tree and the PCoA, it was deemed appropriate to present the results for each species separately. For each species, the corresponding subtree was extracted from the global tree and rooted according to its root in the global tree.

For chromosome painting, seven primary pools were defined. A pool consensus was built for *M*. *balbisiana*, *M*. *itinerans*, *M*. *yunnanensis*, and *M*. *acuminata* subsp. *burmannica*, based on 101, 27, 17 and 18 collected specimens respectively, excluding specimens in marginal positions in diversity trees or with lower SNP numbers. The threshold for identity was set at 80%, meaning that the genotype at a position is validated for the consensus if present in more than 80% of the specimens. The added external reference accessions were used for *M*. *acuminata* subsp. *banksii*, *zebrina*, and *malaccensis*. For each, the two repeats were combined using a 100% identity threshold. Chromosome paintings of all specimens, except those assigned to pool consensus, were then generated to reveal the level of contribution of these primary pools to their genome.

### Musa balbisiana

**Fig 3:**
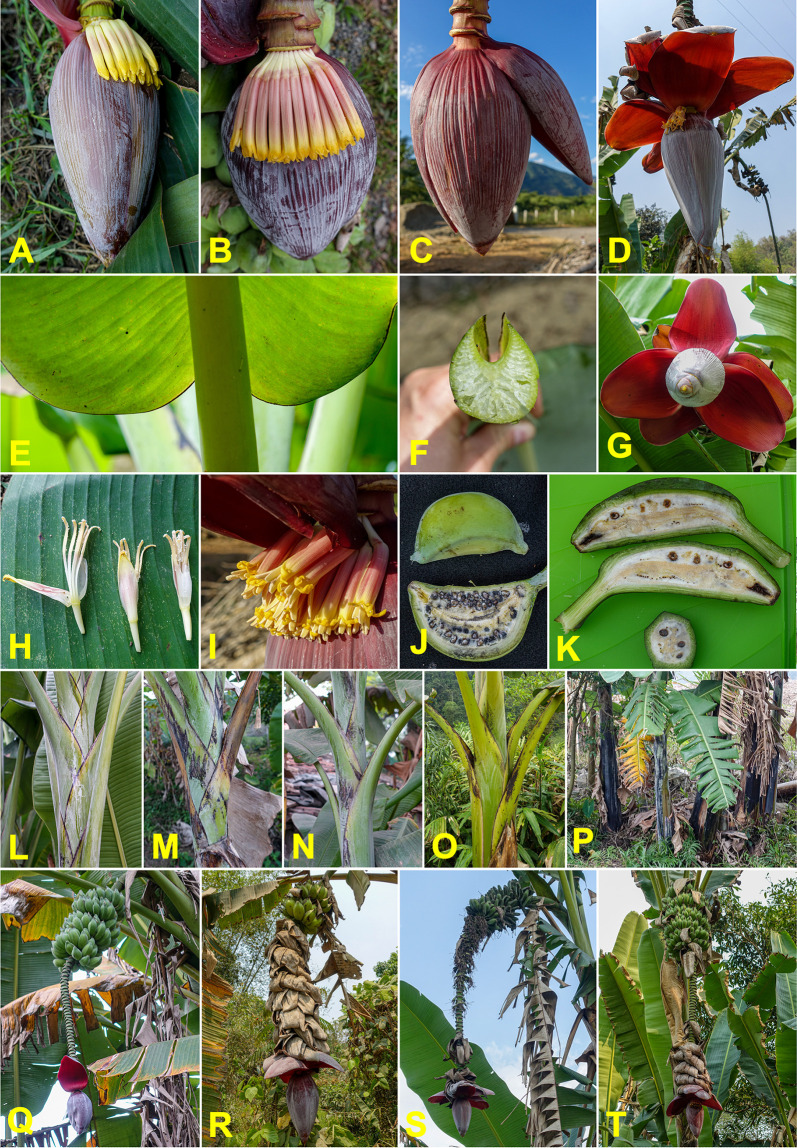
Morphologic diversity of *Musa balbisiana*. (A-D) male bud. (E) the basis of the leaf. (F) petiole canal. (G) bracts. (H-I) male flowers. (J-K) fruits and seeds. (L-P) pseudostem. (Q-T) a complete bunch. Pictures by Matthieu Chabannes—CIRAD.

The species *M*. *balbisiana*, which is abundant throughout the surveyed area, was the most frequently sampled, with 106 specimens collected (**S1 Fig**). The highly distinctive morphological characteristics of this species made identification straightforward in the field. These features include: petiole canal varying from "margins curved inward" to "margins overlapping", black petiole margins, waxy bracts without internal discolouration, absence of bract rolling, bracts often persistent on the male rachis, and fertile fruits with seeds (**Fig 3**).

Almost all these specific characteristics were observed in the collected plants, although some variations were noted such as differences in fruit size or the level of persistence of male flowers and bracts on the rachis. Additionally, pseudostem colours varied from light green, without macules, to black, and with varying abundant wax, a diversity already recognised among the reference specimens in field collections.

Of the 106 specimens collected, 68 were from Vietnam, with two-thirds found in lowland areas, nearly always below 300 m, around Hanoi. The other specimens were observed at elevations over 600 m in western Vietnam, near Laos and Yunnan. In Vietnam, *M*. *balbisiana* was rarely found in wild populations, but was more commonly cultivated in gardens for its pseudostem used as animal fodder, fruits, and seeds, which are medicinally used in alcoholic tinctures. These cultivated plants are clonally propagated, and although their fruits are seeded, the seeds often fail to fully develop and remain soft at maturity [62]. The 26 specimens from Laos and the 12 from Yunnan were collected at medium elevations, around 600–700 m. Wild populations were more abundant towards the West, but the species was also commonly found cultivated in gardens.

**Fig 4:**
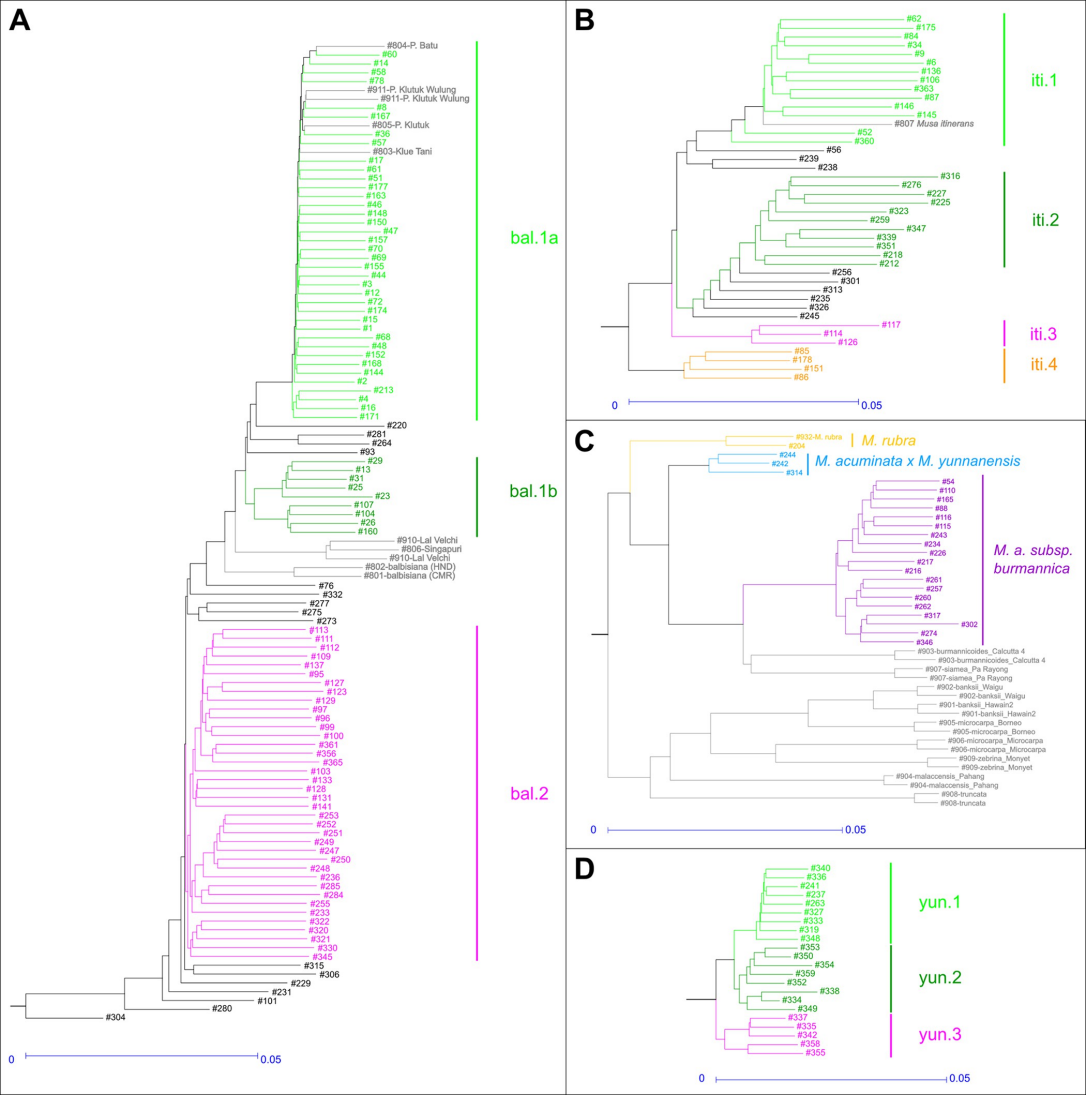
Hierarchical subtrees by species. Subtrees are extracted from the whole tree (**Fig 2C)**, split by species. (A) *Musa balbisiana*, (B) *Musa itinerans*, (C) *Musa acuminata*, (D) *Musa yunnanensis*.

The GBS analysis was conducted on 101 collected plants and eight references (two of which are analysed twice). It revealed a quite structured diversity within *M*. *balbisiana*, which was unexpected for a species reputed to be very homogeneous (**Fig 4A**). Several clusters were identifiable, the most homogeneous was cluster 1a, which included 38 of the 101 specimens and several references: ‘Pisang Batu’, ‘Pisang Klutuk Wulung’, ‘Pisang Klutuk’, and ‘Klue Tani’. Cluster 1b, which included nine specimens, was related to cluster 1a. Cluster 2 had 38 specimens and showed a wider diversity than 1a (**S2 Fig**). Notably, none of the *M*. *balbisiana* references belonged to this cluster. Lastly, several specimens could not be assigned to the above-mentioned clusters (shown in black in the tree), with some, such as #304, being very distant. It is also worth noting that the references of Indian origin (‘Lal Velchi’, ‘Singapuri’), or unknown origin (‘Balbisiana HND’ and ‘Balbisiana CMR’) were not linked to any of the clusters described here, in intermediate position between clusters 1a/1b and cluster 2, indicating a broader genetic diversity in *M*. *balbisiana* species than what was collected in this study area. Therefore, these reference specimens that do not cluster, probably derive from missing wild populations.

This genetic diversity was clearly structured geographically (**S1 Fig**). Clusters 1a and 1b were concentrated in the lowland areas of Vietnam, at a mean elevation of about 100 meters and a median elevation of less than 40 m. In contrast, specimens belonging to cluster 2 were found growing between 200 and 1000 m (with median and mean elevations of more than 600 m). They were mainly found in northern Laos, southern China and northwestern Vietnam, and more rarely farther north in Yunnan.

The heterozygosities of *M*. *balbisiana* specimens were among the highest when compared to the other collected species. Among *Musa* species, many are hermaphrodite with bisexual basal flowers followed by functionally male distal flowers over time. However, several species have basal flowers that are functionally female only, with reduced male organs. These species are therefore mainly cross-pollinated by time shifting. This is the case for *M*. *balbisiana* [63], which is consistent with the observed heterozygosities. However, no incompatibility factors have been found in section *Musa*, so even in the case of unisexual basal flowers, self-pollination is possible by geitonogamy between two inflorescences of the same clump.

**Fig 5:**
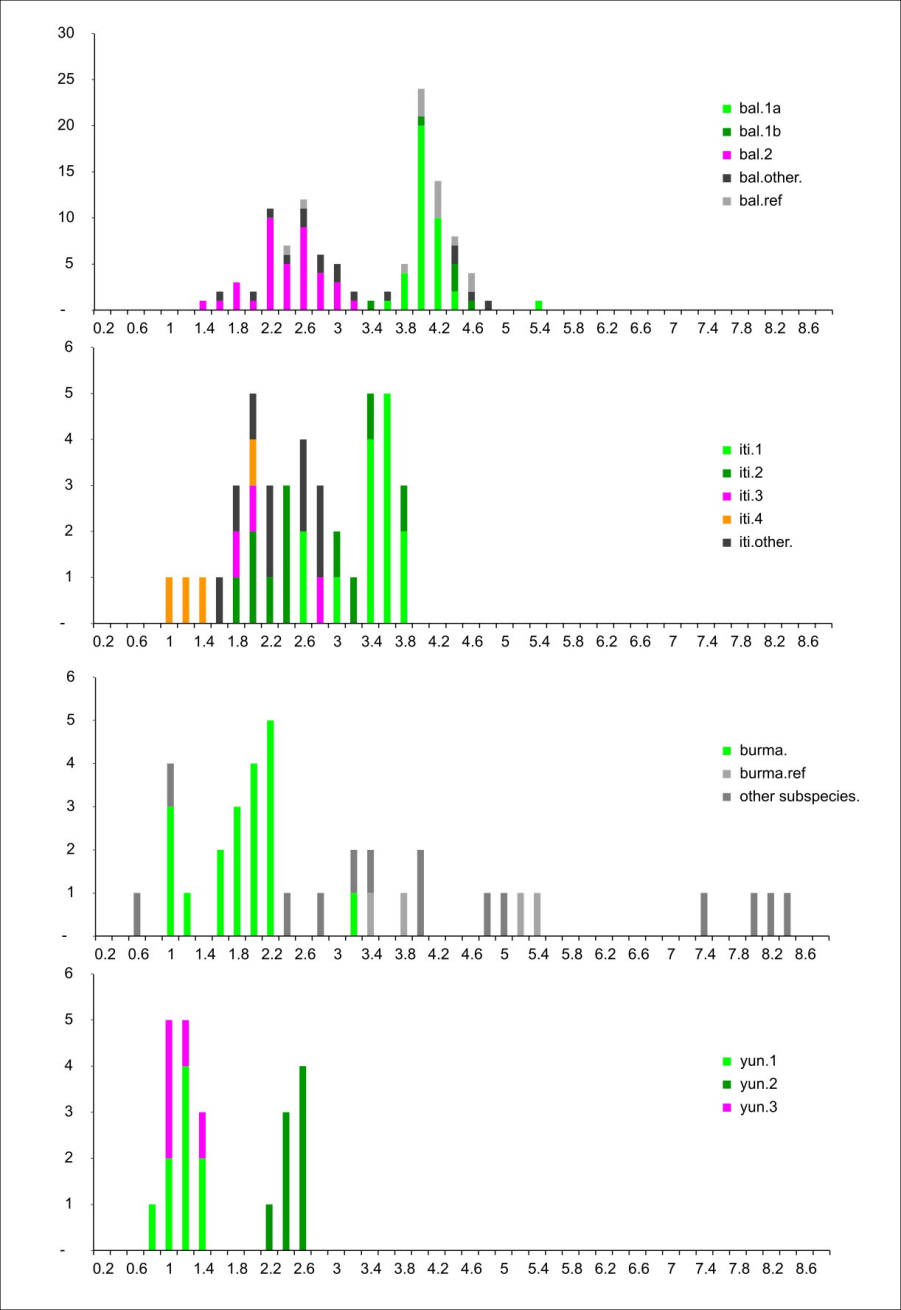
Histograms of heterozygosities by species. Distributed over genomic clusters, unassigned specimens (other), and references (ref.) (Colours according to the legend).

Heterozygosities were higher in clusters 1a and 1b (4.0% on average) than in cluster 2 (2.3% on average) (**Fig 5**). Consequently, a “pole 1” with higher heterozygosities could be identified, including clusters 1a, 1b, and the closely related unassigned specimens, as well as most of the references. Conversely, “pole 2” with low heterozygosity, would include cluster 2, the unclassified samples from China and Laos as well as the two references of unknown origin (‘Balbisiana HND’ and ‘Balbisiana CMR’).

It should be noted that the distinction between the identified clusters, discriminated by only a small number of SNP markers, is not sufficient to propose a subspecies-type botanical division, as is usually agreed for *M*. *acuminata*. This is consistent with the scarce morphotaxonomic differences observed.

The natural range of *M*. *balbisiana* is extensive, covering the entire range of section *Musa*, from India to the Philippines and the islands of nearby Oceania [64]. However, the range of spontaneous forms is much narrower, covering mainland Southeast Asia, from Myanmar, parts of northeast India [65] and the Himalayan foothills, to the provinces of southern China [66]. In the Indian peninsula, the absence of macroglossine bats, the main pollinators of *Musa*, suggests that *M*. *balbisiana* is necessarily cultivated there [67]. Similarly, elsewhere, *M*. *balbisiana* would have been introduced by humans and the observed natural populations would be naturalised forms: Java [64], Sulawesi, Maluku, Lesser Sunda islands [68], Taiwan [69], the Philippines [66], and Papua New Guinea [40, 70]. Indeed, *M*. *balbisiana* is still widely cultivated in gardens, or on small plots, for uses other than fruit [71]. It is particularly resistant to several leaf spots, so its leaves are everywhere favoured and sold for wrapping food or used as plates [72]. The male bud is often used as a vegetable, the pseudostem fibres are used in some regions to make cloth, and various plant parts are known to have medicinal properties [73]. The fruits can be eaten before the seeds ripen and harden. Additionally, some varieties bear fewer seeds, remaining soft at maturity, and can therefore be eaten. In the Philippines, for instance, the variety Pacol displays such partial seed sterility and is cultivated by subsistence farmers [43]. These soft-seeded banana plants may have been selected, leading to a form of domestication where different varieties, vegetatively propagated, are recognized and named [72]. *M*. *balbisiana* is particularly robust and adaptable to conditions quite different from its area of origin. When moved by humans, it can spread over large areas, where crop escapes can then form spontaneous populations.

The study area covers part of the primary endemic area of *M*. *balbisiana* and of the areas of secondary spread. In Vietnam, it was found in compound gardens, in small cultivated plots and much more rarely in natural populations. As previously mentioned, the only wild populations were observed in the northwest of the country [74]. Lê *et al*. [62] identified two types of *M*. *balbisiana* populations in Vietnam: (1) wild, with many seeds, often locally named ‘Chuoi hot rung’; (2) low and soft-seeded, named ‘Chuoi Hot’ or ‘Chuoi Hot Qua Lep’.

We propose that genetic cluster 2 and the few other linked high-elevation specimens (collectively referred to as “pole 2” above) represent the native “wild” compartment of *M*. *balbisiana*. Clusters 1a and 1b, along with the related non-clustered linked specimens, (collectively referred to as “pole 1”) represent a compartment of *M*. *balbisiana*, considered here as "domesticated", in contrast to the previous “wild” compartment. This domesticated pole shows lower overall intra-diversity, which is expected for a plant, at least partially, vegetatively propagated by men. It also displays higher heterozygosity compared to the heterozygosity of pole 2 which is similar in magnitude to that of other wild species (**Fig 5**). This higher heterozygosity can be attributed to the recruitment of alleles from a broader area with significant geographical mixing, coupled with the fixation of rare alleles through vegetative propagation. Spatially, the wild pole 2 is mainly located in the more remote areas of Yunnan and Laos, whereas the domesticated pole 1 is present in the more open areas of Vietnam. Similarly, Mertens *et al*. [75] concluded from comparison based on SSR markers that populations sampled in central and northern Vietnam belong to the native range of *M*. *balbisiana*, while those in southern Vietnam appear to be imported and non-native. It is also worth noting that all the reference specimens, except for ‘Balbisiana CMR’ and ‘Balbisiana HND’, of unknown origin, fall within pole 1. When the origin of these references is known, they are from regions outside the native area of *M*. *balbisiana* and can be considered as references for the domesticated and ubiquitous pole 1. This is also the case for the different *M*. *balbisiana* accessions conserved at the International Musa Germplasm Transit Centre (ITC) *ex situ* collection. According to Mertens *et al*. [76], using SSR marker data, except for one specimen (ITC1527, from Xishuangbanna in southern Yunnan), 27 of the 28 *M*. *balbisiana* accessions at the ITC do not group with native *M*. *balbisiana* populations, which questions their ‘wild’ status.

It is therefore important to enrich the *ex-situ*, *in vivo*, or *in vitro* collections with specimens from the primary zones of the species. Such specimens would serve as true references for the wild pole 2 of *M*. *balbisiana*. The higher intra-diversity observed in pole 2 suggests that these populations could retain some original traits of interest that may have been lost in pole 1 and that could be crucial for future conservation and breeding programmes.

### Musa itinerans

Wild populations of *M*. *itinerans* were frequently found in wild areas, along roadsides and in forests. A total of 35 plants were collected in North Vietnam, 11 in Laos, and 10 in Yunnan (**S3 Fig**).

**Fig 6:**
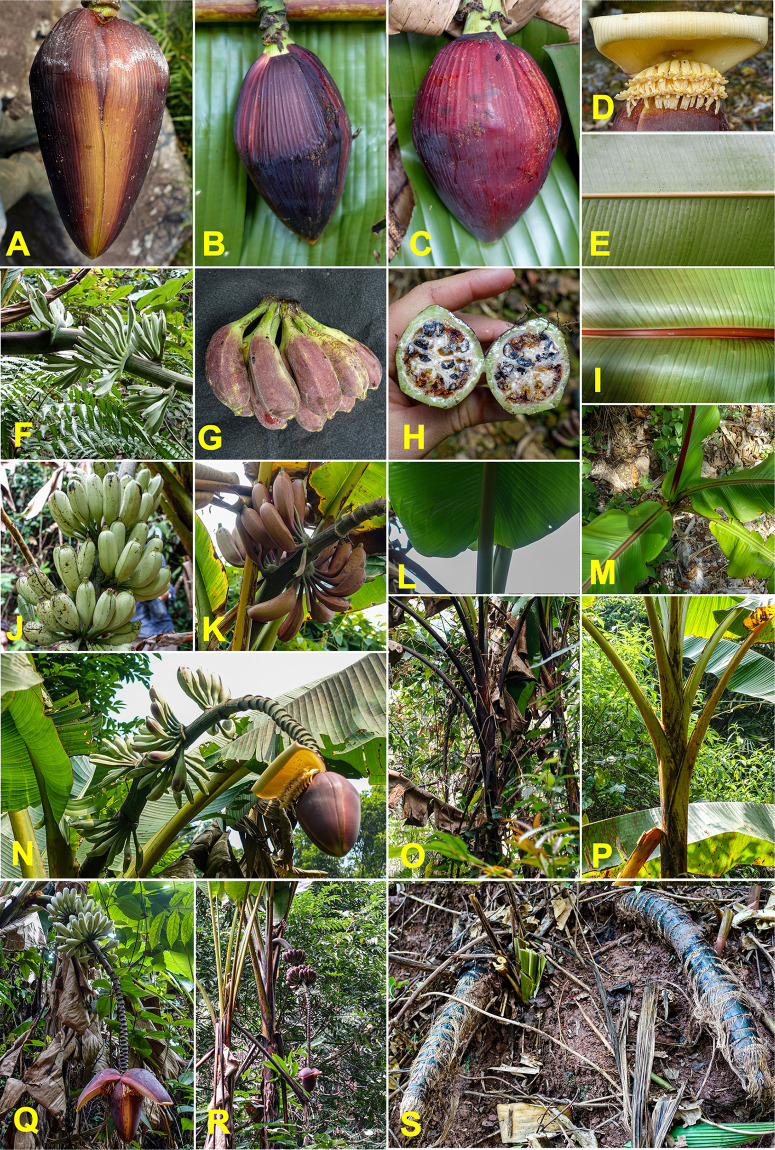
Morphologic diversity of *Musa itinerans*. (A–C) male bud. (D) male flowers. (E, I, L & M) leaves and midrib shape and colour. (F, G, H, J & K) fruits and seeds. (O-P) pseudostem. (N, Q & R) a complete bunch. (S) rhizome. Pictures by Matthieu Chabannes—CIRAD.

All observed plants displayed the distinctive characteristics of the species: a bud with mixed yellow and reddish-brown colours, small fruits with a long pedicel, and suckers branching at a great distance from the mother plant, hence the species name proposed by Cheesman [77] (**S6 Fig**). However, phenotypic variations were noted among the collected specimens. These differences did not challenge the botanical classification as *M*. *itinerans*, which is known to be highly polymorphic [47]. The main variations included the shape and colour of the male bud (**Fig 6A–6C and 6N**), which was more or less ovoid. The fruits were sometimes empty, indicating that the plant is not hermaphrodite (**Fig 6F and 6N**) as previously noted [47, 77]. When pollinated, the fruits were filled with well-formed and complete seeds (**Fig 6H**). Fruits could vary in length but were all located at the end of a characteristic long pedicel. At maturity, the colour of the fruits ranged from light green to brown, pink-red, and purple (**Fig 6G, 6J and 6K**). The bunch position was also variable, often sub-horizontal, sometimes horizontal, or angled (**Fig 6N, 6Q and 6R**). Additionally, some specimens had a black-purple pseudostem, while others had green or yellow pseudostems (**Fig 6O and 6P**).

These populations were found from the lowlands of Vietnam to elevations up to 1600 m in the mountainous areas of North Vietnam and Yunnan, in quite different ecological zones. *M*. *itinerans* is known to form large populations easily, thanks to its spreading rhizomatous root system, and is often a common pioneer species in degraded secondary tropical forests [47]. The species is dispersed across continental Southeast Asia including Yunnan, upper Burma (Myanmar) [77, 78], Laos, Vietnam [62, 74], and North Thailand [64]. In India, the species is confined to the northeast states: Manipur, Mizoram, and Arunachal Pradesh [72], as well as Nagaland [65]. *M*. *itinerans* is also distributed in southern China, including Hainan Island, Guangxi, and Guangdong provinces [79, 80], and extends to Taiwan where several varieties have been described [47]. Thus, the species appears to be widely geographically distributed. The area covered in this study is central to this distribution, and the collected specimens can therefore be considered well representative of the species.

The diversity tree of the 41 *M*. *itinerans* SNP genotypes (plus one reference from the CIRAD collection in Guadeloupe: PT-BA-00223) showed two main clusters, each grouping a core of closely related specimens, designated as iti.1 and iti.2, along with a few other slightly more distant specimens that were (shown in black on the tree, **Fig 4B**). A smaller cluster, iti.3, was still closely related to the first two. Cluster iti.4 was more distinct, indicating intraspecific diversity within *M*. *itinerans*. The high reticulations within the Itinerans group (**Fig 2C**) suggested that clusters iti.1 and iti.2 were partly intermixed. In contrast, the reticulation involving iti.4 showed a slight relationship with the Acuminata pole. Similarly, on the PCoA plan 1–2, the iti.4 specimens appear as the four lowest points of the Itinerans cloud, attracted by the Acuminata pole (**Fig 2B**). In these specimens, the loci specific to *M*. *itinerans* predominate across all the chromosomes, but some loci specific to *M*. *acuminata* are also present. These loci are scattered among the Itinerans loci rather than grouped into continuous segments ruling out recent interspecific hybridisation. All specimens in cluster iti.1 were found in the lowland areas of Vietnam (or in Yunnan, but just north of the Vietnamese border) (**S3 Fig**). The reference from the Guadeloupe collection logically fitted into this group according to its origin to the northwest of Hanoi. The second cluster iti.2 was geographically quite distinct, it included all plants collected in Laos and Yunnan at higher elevations, ranging from 500 to 1000 m, and was absent from Vietnam. The specimens in iti.3 were located in an intermediate area between the previous two, northeast of Dien Bien Phu, near the Chinese border. The specimens in iti.4, genetically more distant, were found in the same region as iti.1, at low elevations around Hanoi in Vietnam. However, it is noteworthy that two iti.1 specimens, were collected from high elevations in western Vietnam and in the north, on the Chinese border. Conversely, two iti.2 specimens were collected at low elevations in the Vientiane region of Laos (**S3 Fig**). This geographical distribution of the iti.1 and iti.2 genomic clusters should thus be interpreted as differentiation by partial isolation rather than adaptive selection to different ecological conditions. Importantly, none of the observed morphological variations correlated with the distribution of individuals across the different genomic clusters. This suggests that the varieties that have been proposed to date, within *M*. *itinerans*, could be just morphological variants without necessarily genetic support.

The distribution of dissimilarities (**S2 Fig**) indicated nearly equal intra-group diversity for iti.1 and iti.2, but both at higher values than those observed within clusters of *M*. *balbisiana*, within *M*. *acuminata* subsp. *burmannica*, or within *M*. *yunnanensis*. These higher diversities might reflect the particularly effective vegetative propagation of this species, which ensures the persistence of various genotypes over time. The observed heterozygosities were on average slightly lower than those for *M*. *balbisiana*, *M*. *itinerans* also having only female basal flowers [47]. Within the species, heterozygosities were higher for iti.1 than for iti.2 (**Fig 5**), displaying a bimodal distribution similar to that of *M*. *balbisiana*.

Although *M*. *itinerans* was not observed in cultivation in the surveyed area, it has been reported that, throughout the area, flower buds, inner pseudostem, and tender inner flower stem are commonly eaten as vegetables, the fruits are used medicinally, and the pseudostem is used as fodder for pigs, buffaloes, or cows [74]. It was also pointed out that among the various sympatric wild *Musa* species that are also gathered, *M*. *itinerans* is particularly valued as a delicate vegetable [46]. In Southwest China, young flowers and young pseudostems form a popular dish offered in local restaurants [81]. Furthermore, Uma [72] describes cultural practices in Northeast India, where local Adi tribes maintain this species at the periphery of their fields or plant stoloniferous suckers in their backyards. The interest of local people in this species for various purposes and its easy vegetative propagation suggest a human contribution to the observed allelic admixture and geographical distribution. Cluster iti.2 likely represents local endemic wild forms of *M*. *itinerans* in remote highland areas, while cluster iti.1, with higher heterozygosities, includes more intermixed forms resulting from contacts and local movements within the broad distribution area of *M*. *itinerans*. It thus encompasses a broader diversity than that present in iti.1, as evidenced by specimens in cluster iti.4.

Like *M*. *balbisiana*, the GBS genotypes show two subsets that differ in their levels of heterozygosity and are geographically disjoint. However, unlike *M*. *balbisiana*, *M*. *itinerans* is primarily a wild-gathered plant, with cultural practices limited to sucker transplantation, which nevertheless implies possible human selection and spread. We may therefore be in an in-between situation where human exploitation of natural populations would already impact the species’ diversity. As with *M*. *balbisiana*, it would be beneficial to enrich reference collections with representatives from the native cluster iti.2.

### Musa acuminata

**Fig 7:**
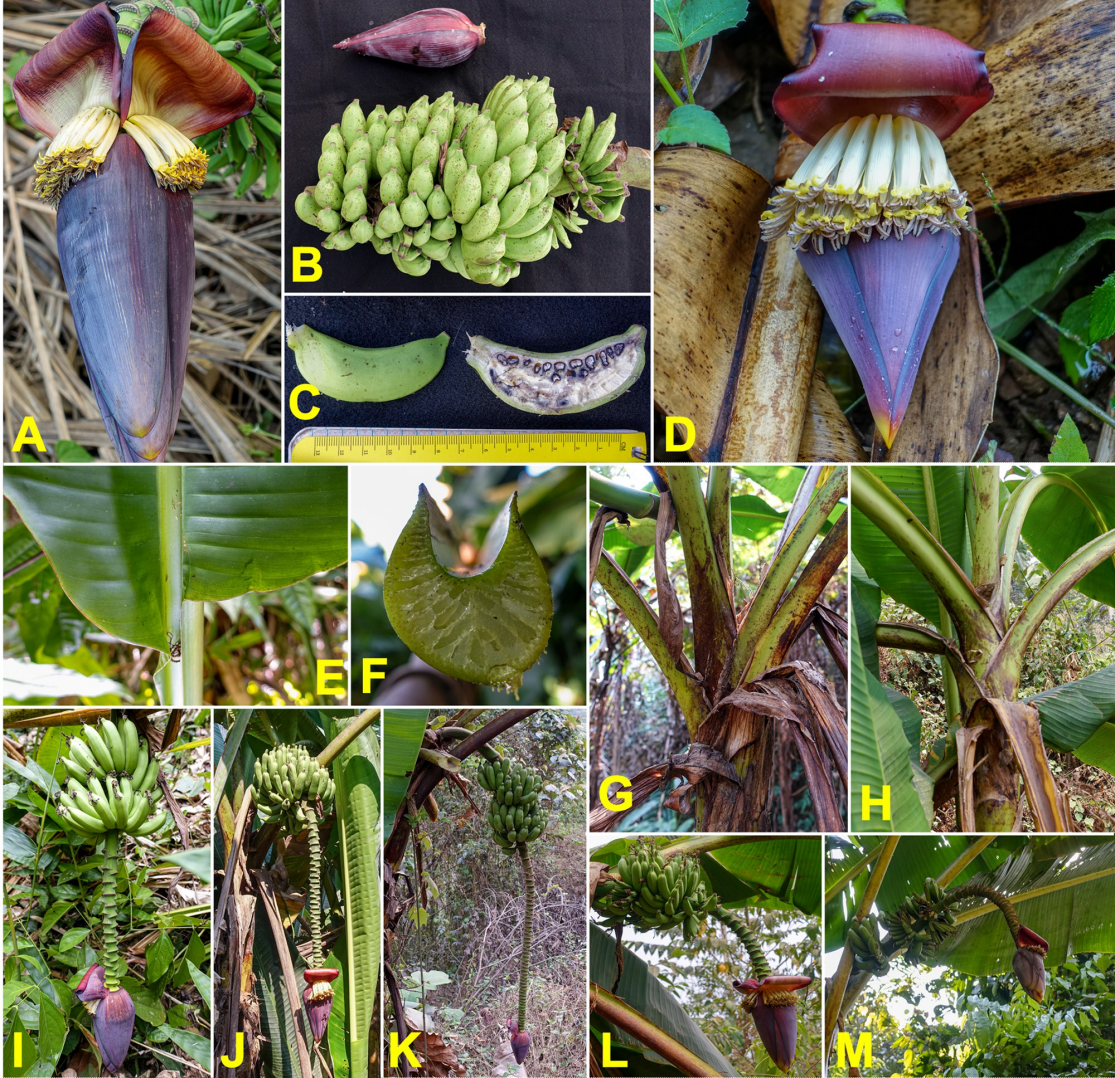
Morphologic diversity of *Musa acuminata*. (A&D) male bud, male flowers. (B-C) fruits and seeds. (E) the basis of the leaf. (F) petiole canal. (G-H) pseudostem. (I-M) a complete bunch. Pictures by Matthieu Chabannes—CIRAD.

Among the specimens collected, 22 samples were assigned to *M*. *acuminata* based on their morphological characteristics. The position of the bunch, often subhorizontal and strongly compact (**Fig 7B, 7I–7M**), along with the colour and shape of the male bud with purple bracts (**Fig 7A and 7D**) and the brown colour of the macules on the pseudostem (**Fig 7G and 7H**), were all indicative of the subspecies *burmannica* or *siamea*. Other notable features included the frequent white discolouration on the inner side of the bract (**Fig 7A**), the general white colour of the male flowers (**Fig 7A and 7D**), and the open shape of the petiole canal (**Fig 7F**).

All plants were collected in the wild, with observation sites concentrated in Laos, particularly in the northern regions, and extending to the adjacent border areas of northeast Vietnam and southern Yunnan (**S4 Fig**). They were almost absent from the entire eastern part of northern Vietnam, as well as from the northernmost surveyed areas in Yunnan. The plants were found at elevations ranging from 200 to 1200 m, mostly around 400 to 500 m.

Among the subspecies previously proposed for *M*. *acuminata*, three have been identified in this geographical area: subsp. *burmannica* [64, 82], subsp. *siamea* N.W. Simmonds [64] and subsp. *burmannicoides* E. De Langhe [83], with the latter being later proposed to be reduced under *burmannica*. The morphological characteristics observed did not allow for a clear assignment of the collected specimens to any one of these subspecies.

Of the 22 specimens collected, 19 were genotyped, along with 18 reference specimens representing the various *acuminata* subspecies. The genotypes of these 19 specimens were quite similar and could be regarded as belonging to the same genomic type (**Fig 4C**). The reference specimens *burmannicoides* ’Calcutta 4’ and *siamea* ’Pa Rayong’ were included in this cluster which was distinct from other *acuminata* subspecies: *banksii*, *zebrina*, *truncata*, and *malaccensis* (**Fig 4C**). The divergences within this cluster were much lower than the divergences with the other subspecies (**S2 Fig**), and the cluster appeared genetically homogeneous, without obvious internal structuring. Heterozygosity was low (1.72% on average) (**Fig 5**). Typically, subspecies of *M*. *acuminata* have female basal flowers [82, 84], however, most of the *burmannica* specimens observed during the Vietnam/Laos/China expedition had hermaphroditic basal flowers. Cheesman [82] also mentioned a specimen from upper Burma, morphologically assignable to *M*. *acuminata*, but with hermaphroditic basal flowers. Nevertheless, some collected specimens, although morphologically identical, had only female basal flowers. Similarly, Joe *et al*. [85] reported female basal flowers in specimens from southern India, but sometimes also hermaphroditic flowers with five functional stamens. This suggests that this trait could easily shift within a population in response, for instance, to ecological conditions.

This hermaphrodite character is in agreement with the observed low heterozygosities. These were much higher in the ‘Calcutta 4’ and ‘Pa Rayong’ references (more than 5%), which raises questions about their status as reference specimens. The heterozygosity of subspecies *banksii* was also low (1.6% on average), a subspecies known to have hermaphroditic basal flowers [82]. Higher heterozygosities were observed in *zebrina* (3%) and reached 7 to 8% in *microcarpa* ’Microcarpa’ and *malaccensis* ’Pahang’.

Failing to distinguish subspecies, we adopted the notion of a subspecies complex, referred to as the Burmannica complex, which groups the three described subspecies: *siamea*, *burmannica*, and *burmannicoides*. These results call for reconsidering the relevance of maintaining three distinct subspecies versus reducing them to three morphological variants of the same subspecies.

Previous genetic studies using molecular markers [31, 86–88], and more recent high-throughput SNP genotyping [30], also highlighted the close proximity of the nuclear, as well as cytoplasmic, genomes of these three subspecies compared to other *acuminata* subspecies.

Additionally, two reciprocal translocations (chromosomes 2/8 and 1/9) provide a specific genetic signature common to the *burmannica*, *burmannicoides*, and *siamea* accessions [89]. Shepherd predicted these translocations using cytogenetic approaches [90], concluding similarly about the similarity of these three subspecies. However, structural chromosome heterozygosity was detected in ‘Pa Rayong’ and ‘Long Tavoy’, indicating a more complicated evolution within the Burmannica complex [91].

The distance of the references ’Calcutta 4’ and ’Pa Rayong’ from the collected specimens (**Fig 4C**) suggests a greater intraspecific diversity within this Burmannica complex, not sampled in this study. This also highlights that the references available in main germplasm collections lack representatives of this group from North Vietnam, North Laos, and Yunnan, and, therefore, do not encompass the overall diversity of this subspecies.

The geographical range covered in this study also extends to Myanmar (then Burma): the area around Myitkyina in the north and further south near Dawei (formerly Tavoy), as well as the coastal islands in the Andaman Sea [64, 82, 83]. In particular, the references ‘Calcutta 4’ and ‘Long Tavoy’ from the Calcutta Botanical Garden are of Burmese origin. The subspecies *siamea* is documented in Thailand but the great variability reported questions about the validity of its subspecies status [46].

This range does not appear to extend westward into the northeastern Indian subcontinent, where, however, several other *Musa* species are attested [92, 93]. Only a few isolated instances of the Burmannica complex are reported in Assam and Meghalaya [72]. However, occurrences of the Burmannica complex are attested on the southwest coast, in the moist evergreen mountain forests of the Western Ghats [94, 95]. It should also be noted that the specimens from the Madras province described by Jacob [96] as *Musa kattuvazhana* (‘forest banana’ in Malayalam), and recently redescribed [97], are native to the Western Ghats. The synonymy of *M*. *kattuvazhana* K. Jacob with *M*. *acuminata* subsp. *burmannica* has been established [85].

Further south, in a small mountainous region in southwest Sri Lanka, a seminiferous banana known locally as ’Unel’ or ’Unakehel’ has been identified as *M*. *acuminata* subsp. *burmannica* [98]. Additionally, specimens of *M*. *acuminata* described in the Andaman and Nicobar Islands [99] as *M*. *kattuvazhana*, have been genomically confirmed as part of the Burmannica complex of *M*. *acuminata* [100].

The spatial discontinuity and distance from the central distribution area, alongside sporadic distribution, as well as the absence of other *Musa* species, suggest local naturalization of introduced plants in the Western Ghats and Sri Lanka. The presence in the Andaman and Nicobar Islands, off the Burmese coast, could imply a transfer to Sri Lanka via the Indian Ocean and a subsequent transfer to the Indian coast. These sporadic and discontinuous occurrences do not support natural dispersal mechanisms, such as bird-mediated seed spread, prompting questions about the people, and their motivations, behind these transfers.

Complete sequencing of various subspecies of *M*. *acuminata* suggests rapid radiation shortly after divergence from *M*. *balbisiana* [29]. The Burmannica complex, positioned at the base of the evolutionary tree, represents a primary form. Later, the dispersal of *M*. *acuminata* subspecies from northwest to southeast Asia led to differentiation through geographic isolation. This differentiation however, is only partial, allowing for intersubspecific hybridisations. For instance, hybrid forms involving subspecies *malaccensis* and the Burmannica complex have been detected by high-density genotyping [30], these wild hybrids originated in northern Thailand, an area where the distributions of the two subspecies overlap.

In summary, *M*. *acuminata* is well-represented in the study area, by forms belonging to the Burmannica complex. Unlike *M*. *balbisiana* or *M*. *itinerans*, the observed range does not cover the entire study area but is restricted to northern Laos and neighbouring regions of Vietnam and Yunnan. Publications of earlier collecting missions extend this range to eastern Myanmar and northern Thailand, but scarcely further. The region centred on upper Laos thus appears to be the botanical core of this complex and the genome common to the collected specimens can be seen as the original genome of this complex. These genotypes are notably absent from reference collections, highlighting the need for their introduction, firstly in the *in vitro* collection of the International Transit Center (ITC). This is especially important as the Burmannica complex is distinct from other *acuminata* subspecies and represents a primitive *acuminata* form ensuring the connection with the other species of *Musa*.

### Musa yunnanensis

The wild species *M*. *yunnanensis* was commonly found as a wild plant, growing abundantly along roadsides, and occasionally cultivated for animal feed and non-fruit uses. We collected 22 specimens in Yunnan Province of China and three specimens in northern Laos, near the Chinese border (**S5 Fig**). The morphological type was easily identifiable according to the morphotype description [101], with some variations in male bud persistence and the presence of withered bracts.

As described by Häkkinen and Wang [102], the species is highly cold-tolerant and occurs exclusively in high-altitude forests up to 2100 m in Yunnan and likely in neighbouring Myanmar. This species thrives in wild populations along roadsides, ravines, and steep slopes, as well as in cultivated populations as animal fodder. Four varieties have been defined within *M*. *yunnanensis* [102]. The *yunnanensis* variety is the common type, about 5 m high with 8-handed bunches. The *caii* variety is smaller, not exceeding 4 m, with 3-handed bunches. The *yongpingensis* variety is the largest, reaching up to 6 m in height, with10-handed bunches. The *jingdongensis* variety is about 4 m high, with 10-handed bunches.

Genome polymorphisms resulting from GBS genotyping of 22 of the 25 analysed specimens clearly distinguished the genome of *M*. *yunnanensis* from that of *M*. *itinerans*, *M*. *balbisiana* and *M*. *acuminata*, with much lower intraspecific diversity than these other species. In the diversity tree as on the factorial plan (**Fig 2B and 2C**), the proximity to specimens of the former *Rhodochlamys* section was notable. Li and colleagues [3] had already shown, based on nuclear ITS markers, the close relationship between *M*. *yunnanensis* and *M*. *ornata*. Despite the low intraspecific variability of *M*. *yunnanensis*, the tree showed three distinct clusters (**Fig 4D**). Cluster yun.1 was found in mid-elevation areas, around 1100 m on average, from Laos to central Yunnan. Cluster yun.2 was related to yun.1 but with slightly wider internal variability, at higher elevations between 1600 and 1800 m. The last cluster, yun.3, included specimens observed only at high elevations, around 2000 m. The heterozygosity of clusters yun.1 and yun.3 was similarly low (1.1 and 0.9% on average, respectively), whereas it was much higher for yun.2 (2.3%) (**Fig 5**). *Musa yunnanensis* has hermaphroditic basal flowers and self-pollination is the main mating system [101]. This would support the low heterozygosity of clusters yun.1 and yun.3 but raises questions about yun.2.

**Fig 8:**
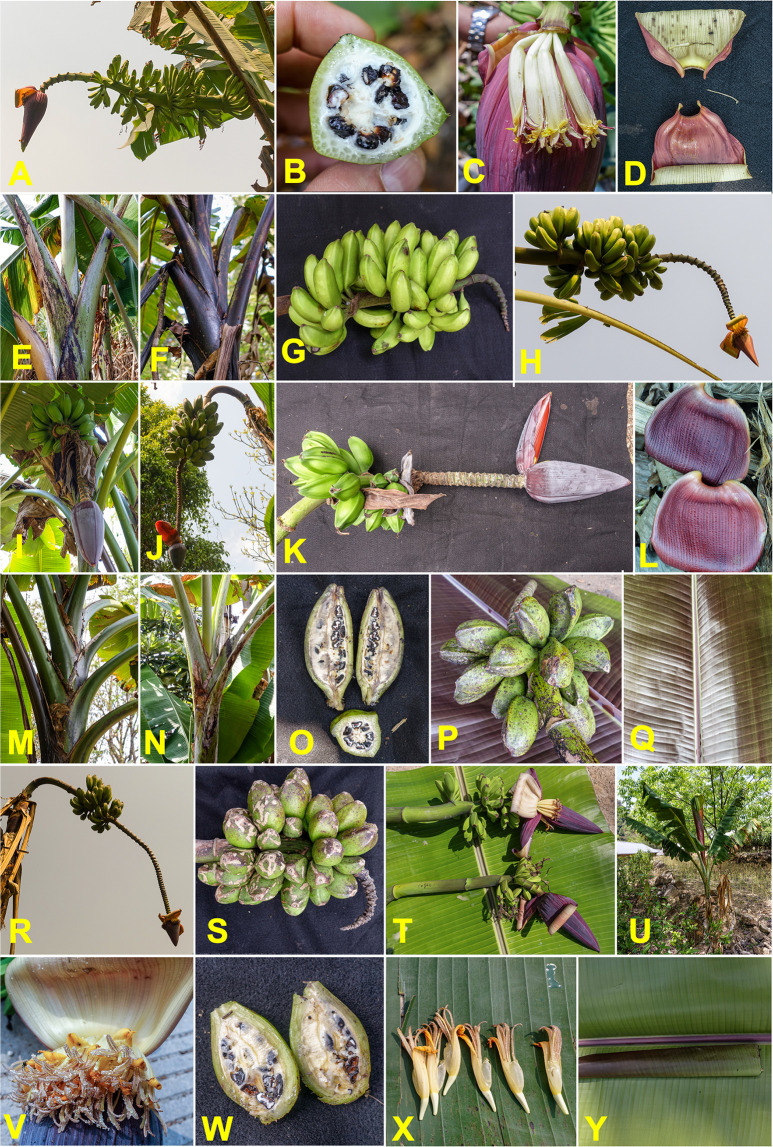
Morphologic diversity of *Musa yunnanensis*. (A–H) *Musa yunnanensis* classical type. (L-Q) *caii* variety. (R-Y) hybrid type. Pictures by Matthieu Chabannes—CIRAD.

Unlike *M*. *balbisiana* and *M*. *itinerans*, the genomic clusters of *M*. *yunnanensis* correlate with morphological types. For yun.1 specimens, the fruits, filled with seeds, are small and swollen at maturity. The bud is tapered, sometimes slightly discoloured, and the bracts are strongly revolute, revealing a white/yellow internal side. The trunk is sometimes entirely black (**Fig 8A–8H**). This type corresponds to the classic morphotype *M*. *yunnanensis var*. *yunnanensis* [102].

The yun.3 cluster included specimens with massive, obtuse buds and globose fruits at maturity (**Fig 8I–8Q**). These specimens can be morphologically assigned to *M*. *yunnanensis* var. *caii*, described by Häkkinen and Wang [102] from specimens typically found up to 2000 m elevation, in mountainous areas where no other *Musa* species grows.

The specimens in the yun.2 cluster displayed combinations of characters assigning them to var. *yongpingensis* [102]. The bunch position is intermediate (horizontal in var. *yunnanensis* and almost vertical in var. *Caii*). The petioles are waxy, with dry papery wings at the base. The male bud has bracts with blue-purple external faces and cream internal faces. The male flowers are cream-yellow with an orange apex of the compound tepal. The male buds often degenerate before fruit maturity. The seeds are tuberculate and large, up to 8-10mm across. The bunch is compact, bearing short fruits with a width roughly half their length and a short pedicel about 1cm long (**Fig 8R–8Y**). Some of the mentioned characters, in addition to the pale purple colour of the dorsal face of young leaves (**Fig 8U and 8Y**), are reminiscent of *M*. *sikkimensis*. This species was initially described in the highlands of Sikkim, on the slopes of the Himalayas in northern India, neighbouring Bhutan [64], Tibet, and possibly Myanmar [103]. The higher heterozygosities of this yun.2 cluster could suggest a more allogamous mating system for this variety.

Chromosome paintings of the three unassigned specimens display a genome combining one haplotype from *M*. *yunnanensis* and one haplotype from *M*. *acuminata* (**S6 Fig**), indicating interspecific F1 hybrid between these species. Their very high heterozygosity, averaging 8.4%, confirms this hybrid nature.

**Fig 9:**
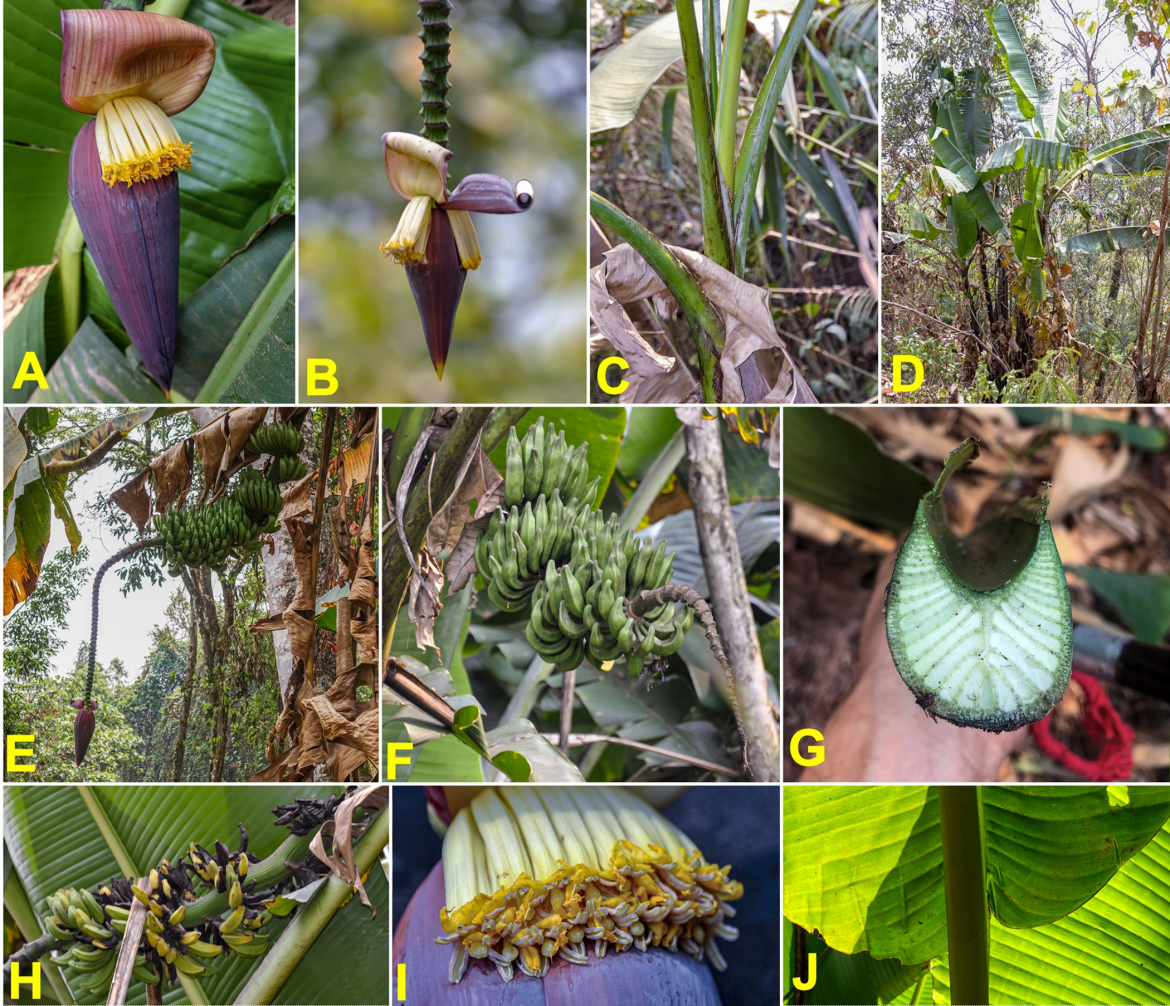
Morphologic characteristics of *Musa acuminata* × *yunnanensis* hybrids. (A–B) Male bud, (C) pseudostem, (D) whole plant in a natural context, (E, F & H) bunch and fruits, (G) petiole canal, (J) the basis of the leaf. Pictures by Matthieu Chabannes—CIRAD & Gabe Sachter-Smith.

The phenotype of these interspecific hybrids was reminiscent of any seminiferous *Musa* section species, with a subhorizontal bunch and numerous hands with small fruits (**Fig 9**), but with characteristics specific to both *M*. *yunnanensis* and *M*. *acuminata*, which led to the assumption of a possible hybrid form when collected in the field. A few rare traces of pollen were found on the flowers and vestiges of seeds were observed inside the fruits, but these seemed aborted and non-functional. The hybrid was therefore most likely sterile or had very low male fertility. The fruits showed no indication of parthenocarpy.

These specimens were found in wild conditions, with two specimens in Laos, at 1250 m elevation, and the third, at 550 m elevation, in China. While the first two were close enough to potentially represent the same population, the third was over 80 km away, suggesting at least two independent events. This indicates that natural interspecific hybridisation is not exceptional. These hybrids were collected in areas where wild populations of the two parent species coexist, specifically at the southern edge of *M*. *yunnanensis* range, corresponding to yun.1 area. Thus, it is likely that the *M*. *yunnanensis* genome contributing to these hybrids comes from the yun.1 cluster.

### Species of the former section *Rhodochlamys*

Several representatives of the ornamental species from the former section *Rhodochlamys* were observed, in Laos and China (**S7 Fig**). The classical types, *M*. *ornata* and *M*. *velutina*, were cultivated in gardens. In contrast, *M*. *rubinea* was found growing spontaneously in the forest. *Musa rubra* (synonym *M*. *laterita*) was observed only once, in cultivation, in southern Laos, near Thailand, where it was said to originate. However, a farmer in Laos indicated that he saw this plant, known as ’Kuay Ka Chung’, growing wild deep in the forest, far from his farm, but the collecting team could not locate any wild populations.

Note that *M*. *rubra* is often referred to as *M*. *laterita*. Cheesman [104] published *M*. *laterita* as a new species from seeds sent from Myanmar while recognising its proximity to *M*. *rubra*. This morphological and genetic similarity was confirmed when Joe and colleagues [5] reduced *M*. *laterita* to be conspecific with *M*. *rubra*.

Genomic characterisation showed that the specimens from the former section *Rhodochlamys* do not form a homogeneous group. In the diversity tree (**Fig 2C**), a first group, which included the species *M*. *ornata*, *M*. *velutina* and *M*. *rubinea*, was distinct from *M*. *acuminata*, *M*. *balbisiana* and *M*. *itinerans*. This group was compact showing much lower intraspecific diversity compared to the three previously mentioned species. As noted earlier, *M*. *yunnanensis* is closely related to this group.

In contrast, the species *M*. *rubra* (both the field specimen and the collection reference), which is morphologically similar to *Rhodochlamys* species in its erect inflorescence, was linked to *M*. *acuminata* (**Fig 2B and 2C**). Within *M*. *acuminata*, *M*. *rubra* was closer to subsp. *burmannica* (**Fig 4C**) than to the other *acuminata* subspecies. Previous studies emphasised the morphological and genomic proximity of *M*. *laterita* to *M*. *acuminata* [3, 86, 105]. Based on the chloroplast genome, *M*. *laterita* was proposed to be a hybrid between a species from section *Rhodochlamys* and *M*. *acuminata*, with the latter providing the maternal material [12]. Our GBS data on the nuclear genome showed highly homozygous chromosomes for *M*. *rubra*, with a significant number of alleles specific to *M*. *acuminata* subsp. *burmannica*, interspersed among alleles specific to other primary pools. For example, on chromosome 6, (**S8A Fig**), *M*. *rubra* showed a pattern distinct from a pure *burmannica* specimen (**S8B Fig**). Therefore, *M*. *rubra* cannot be interpreted as a direct hybrid with *burmannica* but rather as a closely related form, sharing a common ancestor that provided their similar plastome. If *burmannica* is a primitive form within *M*. *acuminata* [29], it makes sense that *M*. *rubra*, as a close relative to *M*. *acuminata*, shares more specific alleles with *burmannica* than with other *acuminata* subspecies.

*M*. *rubra/M*. *laterita* is primarily known worldwide as an ornamental plant [106]. However, this species is also found naturally in South-western Myanmar [107] and in northern and western Thailand [46]. Additionally, it has been reported as wild only in Manipur and Mizoram in North-East India [5]. The range of *M*. *rubra* thus overlaps with that of *M*. *acuminata burmannica*; their joint occurrence in the same geographical area suggests that this region is likely the origin of their common ancestral forms.

## Conclusion

Our study confirms mainland Southeast Asia as a primary centre of diversification for the section *Musa*. The main species present in the region exhibit highly contrasting distributions. *M*. *yunnanensis* is localised in the specific ecologies of the Yunnan highlands. Within the species *Musa acuminata*, only the Burmannica complex is present. It is endemic to upper Laos but has occasionally been transferred over long distances. *Musa itinerans* has a strong presence in all three countries, with a much wider distribution throughout Southeast Asia. *Musa balbisiana* is even more widely distributed and is now found in all areas with a Musa-friendly ecology, posing challenges in distinguishing spontaneous forms from feral ones.

Genomic characterisation has shown intraspecific diversity in all species studied. This diversity does not appear to be correlated with morphological diversity, except in the case of *M*. *yunnanensis*. Interestingly, these genomic data have shown the existence of hybrid forms between the *M*. *acuminata* and *M*. *yunnanensis* species. Such spontaneous hybridisations between wild species, without human intervention, to our knowledge, had never been documented before. They attest to the possible spontaneity of the interspecific hybridisations that are inferred for the early phases of cultivated banana domestication.

Addressing intraspecific genetic diversity has revealed diversity structures that differ in internal variability and levels of heterozygosity. These structures are also geographically organised, but with a configuration that seems, at least for *M*. *balbisiana* and *M*. *itinerans*, to be more related to human factors than to environmental constraints. The conjunction of these elements raises questions about anthropogenic impacts on the organisation of plant diversity. Indeed, these *Musa* species, although not cultivated as such, appear to be diversified and dispersed geographically by human agency. The exploitation by local people of the natural populations, as observed for many CWR in the world [108–111], already impacts on the species’ diversity. It should be stressed that uses other than fruit consumption, which is now the almost exclusive use, were certainly key drivers in the early stages of *Musa* domestication, as was likely also the case for other important crop species.

What is observed here for these *Musa* species might be representative of the processes involved in early domestication within the large family of bananas cultivated today. These four species illustrate various stages of the process. *Musa yunnanensis* is a typical wild form, but already used as livestock fodder. *Musa acuminata* subsp. *burmannica* is also always found in wild conditions; its geographical distribution is still limited, but sporadic dispersal spots are probably associated with human movements. *Musa itinerans*, which has a wide distribution, is not cultivated but is subject to intense human exploitation of natural populations, with minimal agricultural practices such as transplantation of suckers and, therefore, possible selection for particular traits. *Musa balbisiana* is at the most advanced stage of the process. The species can be considered domesticated for its various uses, resulting in a very wide distribution and diversity strongly impacted by human activities. These results lead to revisiting the boundaries between the notions of wild, cultivated, and domesticated bananas, referring to the notion of ‘cultiwild’ as previously proposed by De Langhe and colleagues [112], and defined as morphogenetically wild plants cultivated and translocated by people [113].

It is also important to emphasise that the diversity present in major *ex situ in vitro* or field collections is not representative of the diversity observed in nature. Although they are the primary material for diversity studies and breeding programmes, these collections represent a truncated and biased picture of the whole diversity of *Musa* species. In their respective countries, vegetative materials and/or seeds of some of the specimens sampled were collected to complement and enrich existing collections (Vietnam and China) or to establish a new collection (Laos). However, the field collections are often vulnerable and subject to environmental and economic uncertainties. Therefore, backup conservation of the most original genotypes in other structures is a priority. This requires international coordination and the signing of agreements in compliance with the Convention on Biological Diversity, the Nagoya Protocol, and the International Treaty on Plant Genetic Resources for Food and Agriculture. Moreover, the intraspecific diversity observed for all species also argues for *in situ* management of the diversity of wild forms that habitat fragmentation and gene-flow restriction are threatening. At the very least, it is urgent to develop, maintain, and share information systems that specify the location, status, and risk level of these populations.

Lastly, further collection missions should be carried out in currently less well-known areas in Southeast Asia. This is of the highest priority, as widespread deforestation in many of these areas is leading to the inexorable destruction of unique *Musa* diversity.

## Supporting information

S1 Table List of all collected and analysed samples used in this study. (NumID: study identifier. ID VCF: sample identifier in the original VCF file (where #1 and #2 stand for first and second GBS batches). Former Section: botanical section. Former botanical section (before Häkkinen 2013 merge). Section: present botanical section. Species. Subspecies. Cluster or ’name’: NJ tree cluster or reference name. CRBPT code: accession code in the CRB-PT collection (CIRAD Guadeloupe) where the reference specimens are coming from. Country. Latitude: decimal latitude. Longitude: decimal longitude. Elevation in m. iNaturalist URL: when observations have been posted to iNaturalist, their URL. Valid SNPs: number of valid SNP for this accession. Mean depth: mean of the depths on all the valid SNPs. H%: heterozygosity).
(XLSX)

S1 File GBS-Div software description and functionalities. (DOCX)

S1 Fig Map of all *Musa balbisiana* specimens. Colours by genomic clusters according to the legend (other: not assigned to clusters, ng: not genotyped). Shifting of overlapping points using QGIS internal displacement option. Boxplot of elevations by genomic clusters. Terrain data sourced from OpenStreetMap. Map projection: WGS 84.
(TIF)

S2 Fig Distribution of pairwise dissimilarities by species. For the whole sample (dotted line) and each genomic cluster (line colour according to the legend).
(TIF)

S3 Fig Map of all *Musa itinerans* specimens. Colours by clusters according to the legend (other: not assigned to clusters, ng: not genotyped). Shifting of overlapping points using QGIS internal displacement option. Boxplot of elevations grouped by clusters. Terrain data sourced from OpenStreetMap. Map projection: WGS 84.
(TIF)

S4 Fig Map of all *Musa acuminata* specimens. Colours by clusters according to the legend (ng: not genotyped). Shifting of overlapping points using QGIS internal displacement option. Boxplot of elevations grouped by clusters. Terrain data sourced from OpenStreetMap. Map projection: WGS 84.
(TIF)

S5 Fig Map of all *Musa yunnanensis* specimens. Colours by clusters according to the legend (ng: not genotyped). Shifting of overlapping points using QGIS internal displacement option. Boxplot of elevations grouped by clusters. Terrain data sourced from OpenStreetMap. Map projection: WGS 84.
(TIF)

S6 Fig Chromosome painting for the 11 chromosomes of a *M*. *acuminata* × *yunnanensis* hybrid (#314). Colours are assigned to primary pools according to the legend.
(TIF)

S7 Fig Representatives of the former *Rhodochlamys* section. (A) *Musa rubra* (= ex *Musa laterita*), (B) *Musa ornata*, (C) *Musa velutina*, (D) *Musa rubinea*
(TIF)

S8 Fig Distribution of allelic ratios for *M*. *rubra* & *M*. *acuminata* subsp. *Burmannica*. Allelic ratios (0,5 for heterozygotes, 1 for homozygotes) along chromosomes (e.g., chromosome 6) at specific primary pool loci: (A) the collected specimen of *M*. *rubra* (#204) and (B) a specimen of *M*. *acuminata* subsp. *burmannica* (#217), for comparison. Colours of primary pools according to the legend.
(TIF)
