# Supplementary material for: Musa species in mainland Southeast Asia: From wild to domesticate
Source: PLoS One. 2024 Oct 2;19(10):e0307592. doi: 10.1371/journal.pone.0307592 (PMC11446428; doi:10.1371/journal.pone.0307592)
Supplement: S1 File — (DOCX) [file pone.0307592.s002.docx]

**Supplementary file: GBS-Div software**

The GBS-Div software, developed within the UMR CIRAD/AGAP, provides various functions necessary for the analysis of SNP polymorphisms and can handle populations with various levels of ploidy, whereas other available software generally only handles diploid individuals.

GBS-Div explores VCF files of SNP polymorphisms observed on a set of individuals. It first proposes a series of filters to clean the data and provides statistical indicators for evaluating the effect of these filters.

The distributions of its SNP allele frequency ratios for each individual are used to infer the actual ploidy level. This inference allows genotype calling at each SNP on real ploidy levels and not on an implicit diploidy or ploidy fixed *a priori*, as the few software supporting polyploidy do.

It also addresses possible variations in ploidy levels between chromosomes or even within a chromosome by segmental duplications, events observed in bananas. The distributions of allelic ratios for a given SNP theoretically have central values of 0.5 for heterozygous sites in diploid (1/2 - 1/2 distribution) and 0.66 for a triploid (2/3 - 1/3 distribution). For tetraploids, the heterozygosities are either centred on 0.5 (2/4 - 2/4 distribution) or 0.75 (3/4 - 1/4 distribution). The ratios for homozygous sites are 1 in all cases. Sequencing errors, although rare, and the randomness of the sampling after amplification lead to deviations from these pivotal values, resulting in binomial distributions around these theoretical peaks. GBS-Div assesses these distributions and provides statistical thresholds for inferring ploidies at a given locus. The representation of the estimated ploidies at each locus along the chromosomes, thereby drives the inference of the ploidy of that chromosome, with possible segments of different ploidies on the same chromosome. These local ploidies are recorded in a dedicated file, which will be used for genotype calling based on the actual local ploidies (between x and 4x).

Dissimilarity matrices can be computed for the whole genome, on a particular chromosome, or even on a chromosome segment. The dissimilarity between pairs of individuals is calculated as the ratio of the number of identical alleles (0, 1, or 2) to the total number of SNPs present for the two individuals. The resulting files can be used directly in DARwin (Perrier and Jacquemoud-Collet, 2006), another software package developed by the same team that offers methods for dissimilarity matrix analysis, tree representations, robustness estimation, tree comparison etc.

When several representatives of the same group are available, it may be appropriate to merge them into a synthetic individual that best characterises the group. A consensus procedure is proposed to infer this standard representative. For a given position, a genotype is retained as the consensus genotype if the position is present in a sufficient number of individuals (defined by a configurable threshold) and if this genotype is the most frequent (with a configurable majority rule from 0.5 to 1) for the retained positions.

A group of tools in GBS-Div deals with 'chromosome painting', aiming to identify segments along the chromosomes with determinable ancestral origins. In the first step, from individuals considered representative of the ancestral pools, or their consensus, the loci that discriminate each pool from all the others are screened. To do this, each pool is contrasted against all others, to identify discriminant SNPs. which are loci homozygous for one allele in all individuals in the pool and homozygous for the alternate allele in individuals in all other pools. This very stringent definition limits the number of effective SNPs but ensures that discrimination is focused on specific mutations and excludes any partial similarities due to shared inheritance for these ancestral pools. This stringency can be relaxed by accepting that a few individuals in a group, with a configurable number, have a different genotype and/or by accepting missing individuals in the groups, with a configurable rate.

A function then applies these 'one versus all' contrasts to all individuals in the population. For an individual, the number of alleles specific to the pool is counted at each position declared discriminant of the pool. The representation tool displays, for an individual, the average of these counts on all discriminating positions within a user-defined length window along a chromosome.

It is also possible to merge all the contrasts for a synthetic visualisation of the contribution of these pools to the genome of a particular individual. An interactive graphic tool allows the user to design an optimal segmentation of the chromosomes, assigning to each segment the colour of its ancestral pool. This segmentation is recorded in a specific file and a chromosome painting function allows the display of mosaics of the inferred ancestral contributions for the 11 chromosomes. Note that the GBS method of sequencing very short reads does not give access to phased data; the choice of the phases for chromosome painting remains arbitrary. However, it can be guided by parsimonious criteria, with the solution minimising the number of crossing-over being *a priori* the most probable.

Finally, a series of tools are dedicated to the management of VCF files, such as removing indels and illegal characters, merging multiple VCF files, extracting subsets of individuals, and exporting to other formats.

GBS-Div is not open-source but it is provided free for use in research and education. GBS-Div can be downloaded from: <https://darwin.cirad.fr/gbsdiv.php>.
