# Supplementary figures and images for: Musa species in mainland Southeast Asia: From wild to domesticate
Source: PLoS One. 2024 Oct 2;19(10):e0307592. doi: 10.1371/journal.pone.0307592 (PMC11446428; doi:10.1371/journal.pone.0307592)

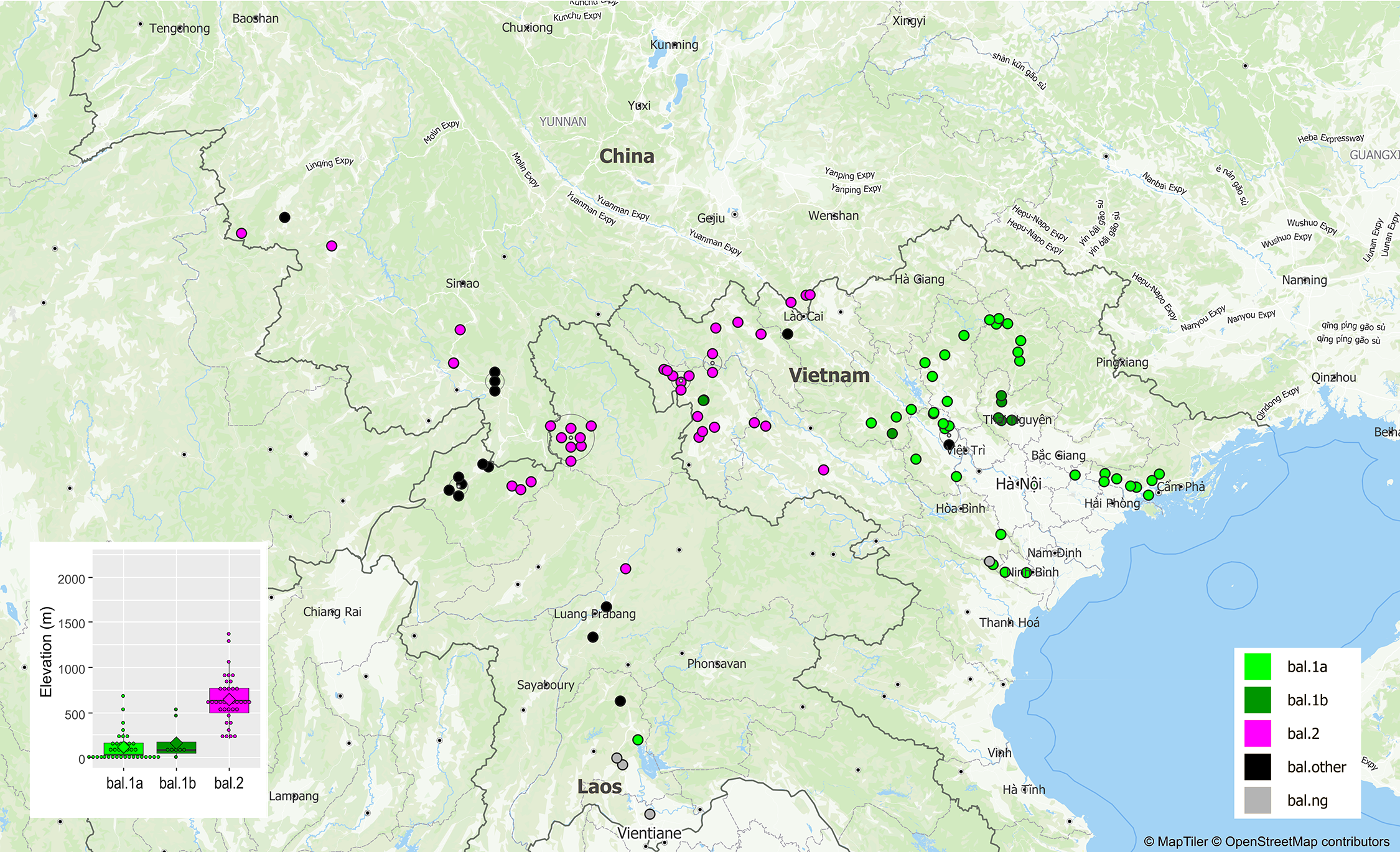

Supplement: S1 Fig — Colours by genomic clusters according to the legend (other: not assigned to clusters, ng: not genotyped). Shifting of overlapping points using QGIS internal displacement option. Boxplot of elevations by genomic clusters. Terrain data sourced from OpenStreetMap. Map projection: WGS 84. (TIF) [file pone.0307592.s003.tif]

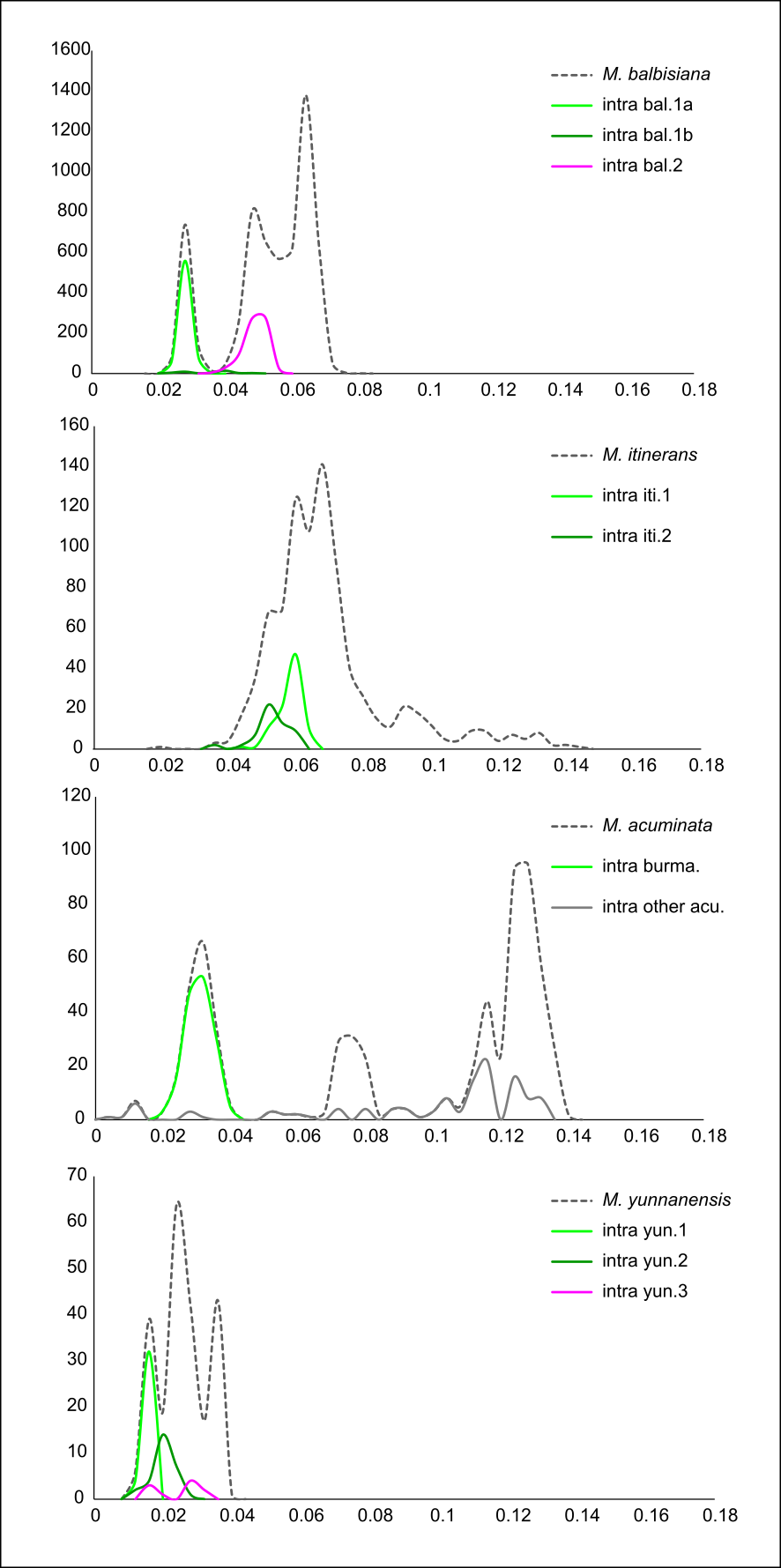

Supplement: S2 Fig — For the whole sample (dotted line) and each genomic cluster (line colour according to the legend). (TIF) [file pone.0307592.s004.tif]

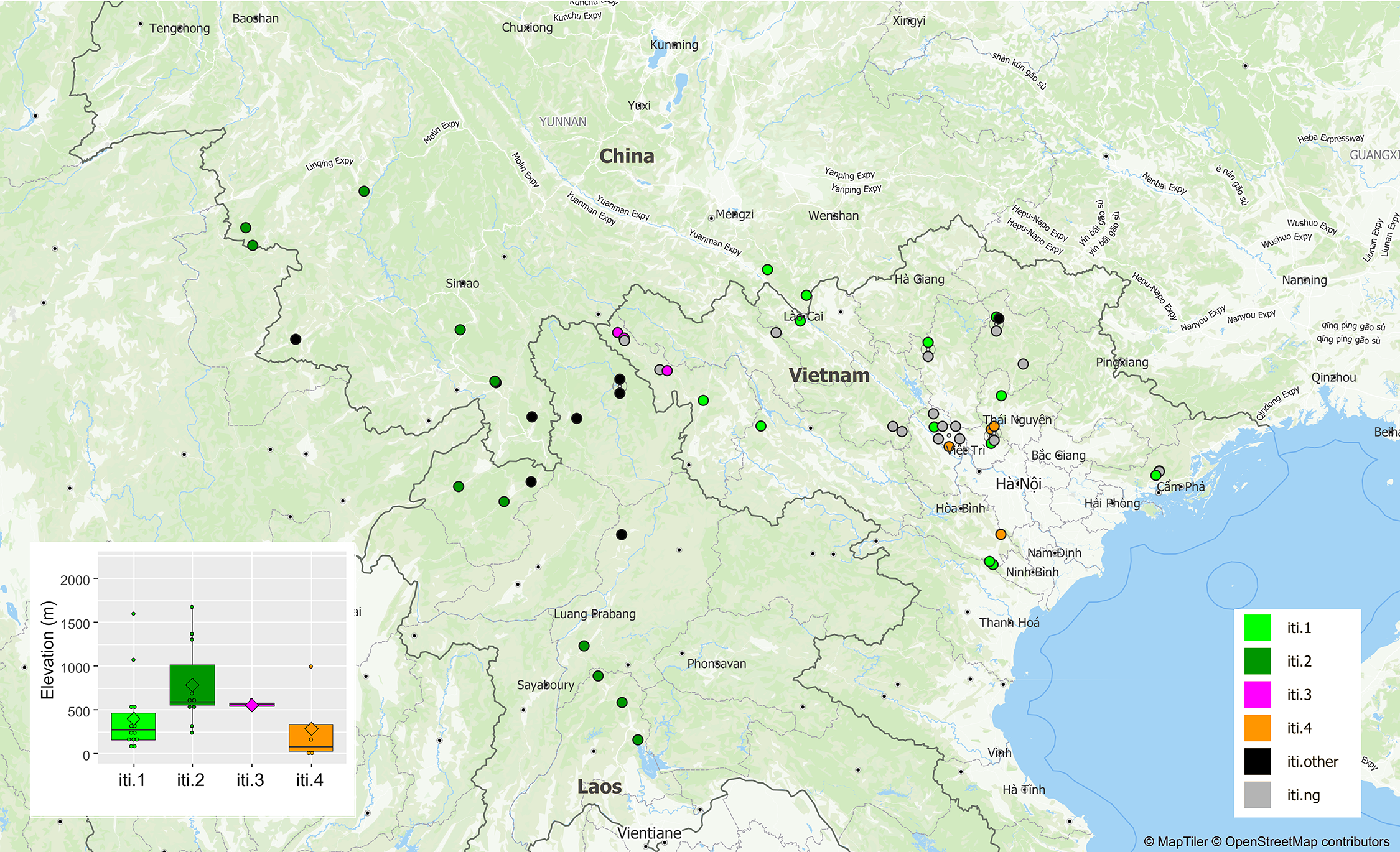

Supplement: S3 Fig — Colours by clusters according to the legend (other: not assigned to clusters, ng: not genotyped). Shifting of overlapping points using QGIS internal displacement option. Boxplot of elevations grouped by clusters. Terrain data sourced from OpenStreetMap. Map projection: WGS 84. (TIF) [file pone.0307592.s005.tif]

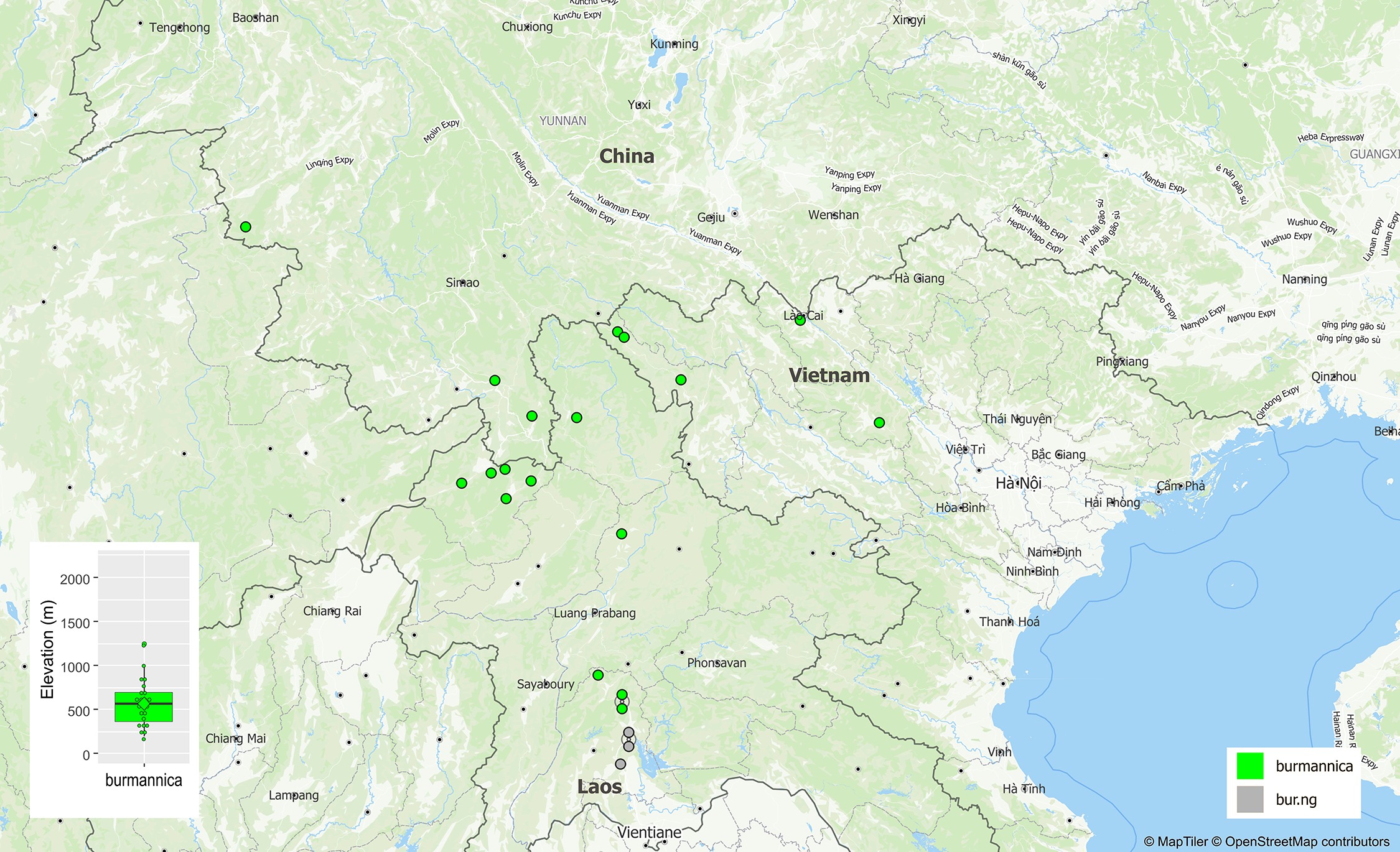

Supplement: S4 Fig — Colours by clusters according to the legend (ng: not genotyped). Shifting of overlapping points using QGIS internal displacement option. Boxplot of elevations grouped by clusters. Terrain data sourced from OpenStreetMap. Map projection: WGS 84. (TIF) [file pone.0307592.s006.tif]

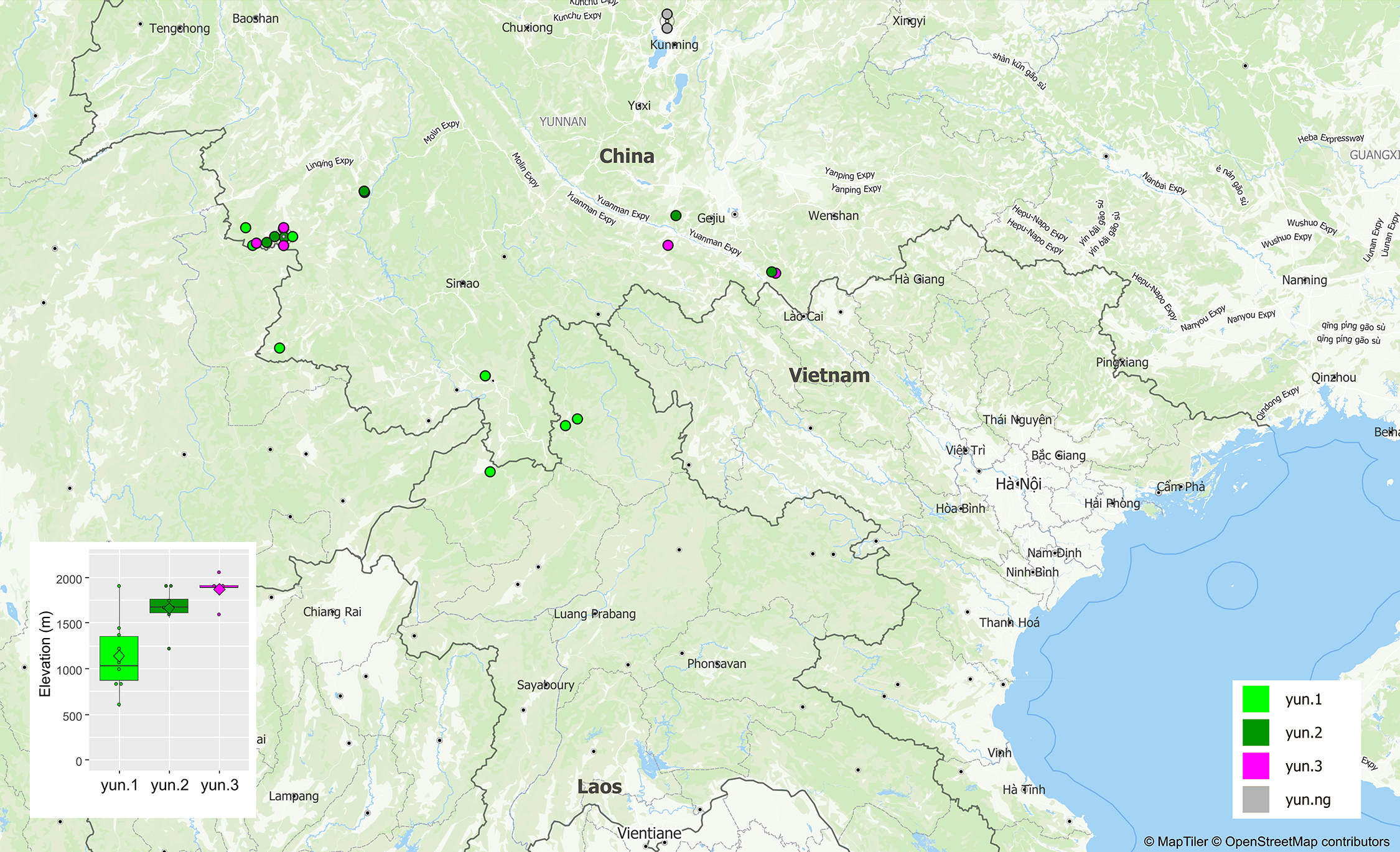

Supplement: S5 Fig — Colours by clusters according to the legend (ng: not genotyped). Shifting of overlapping points using QGIS internal displacement option. Boxplot of elevations grouped by clusters. Terrain data sourced from OpenStreetMap. Map projection: WGS 84. (TIF) [file pone.0307592.s007.tif]

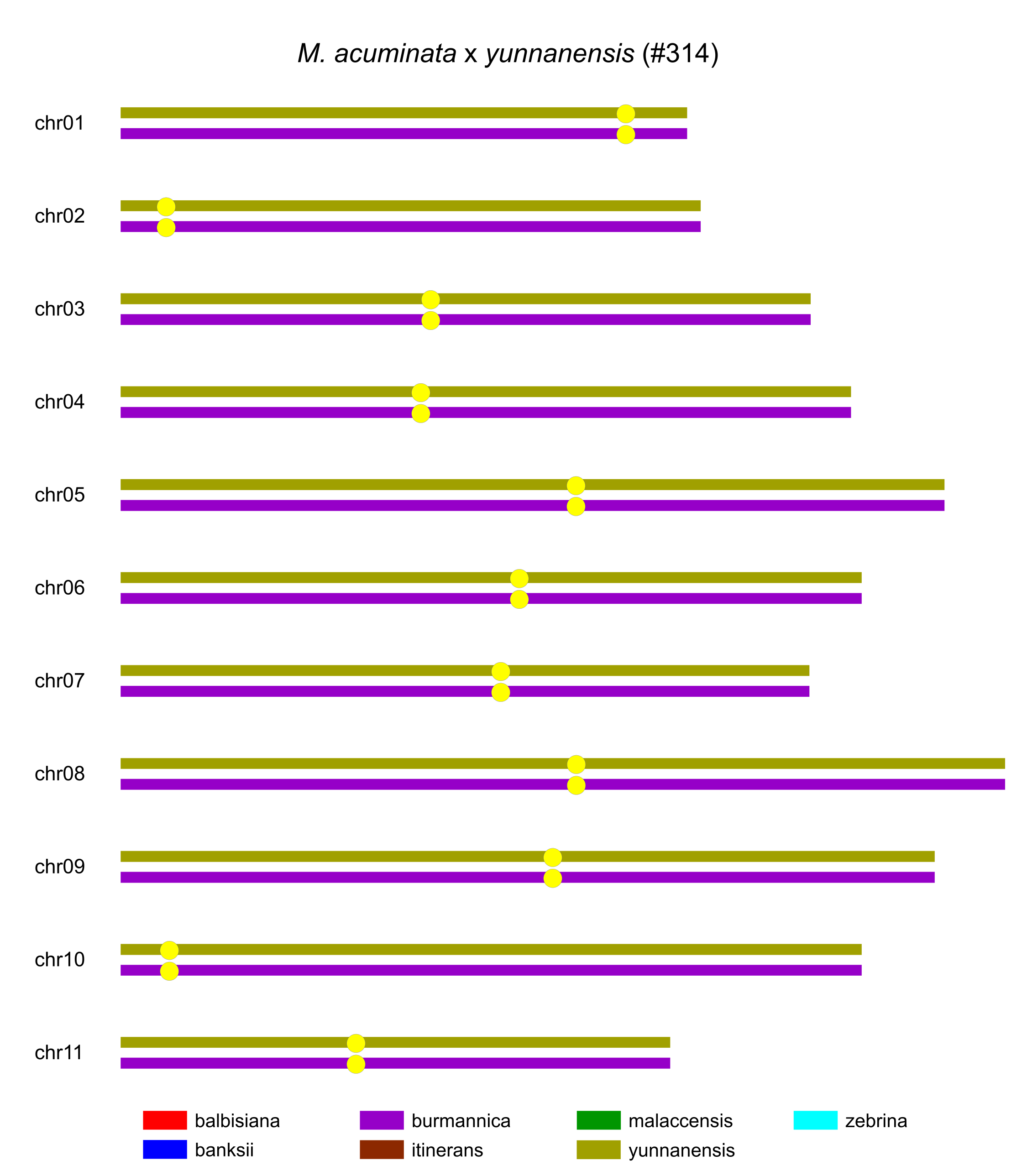

Supplement: S6 Fig — Colours are assigned to primary pools according to the legend. (TIF) [file pone.0307592.s008.tif]

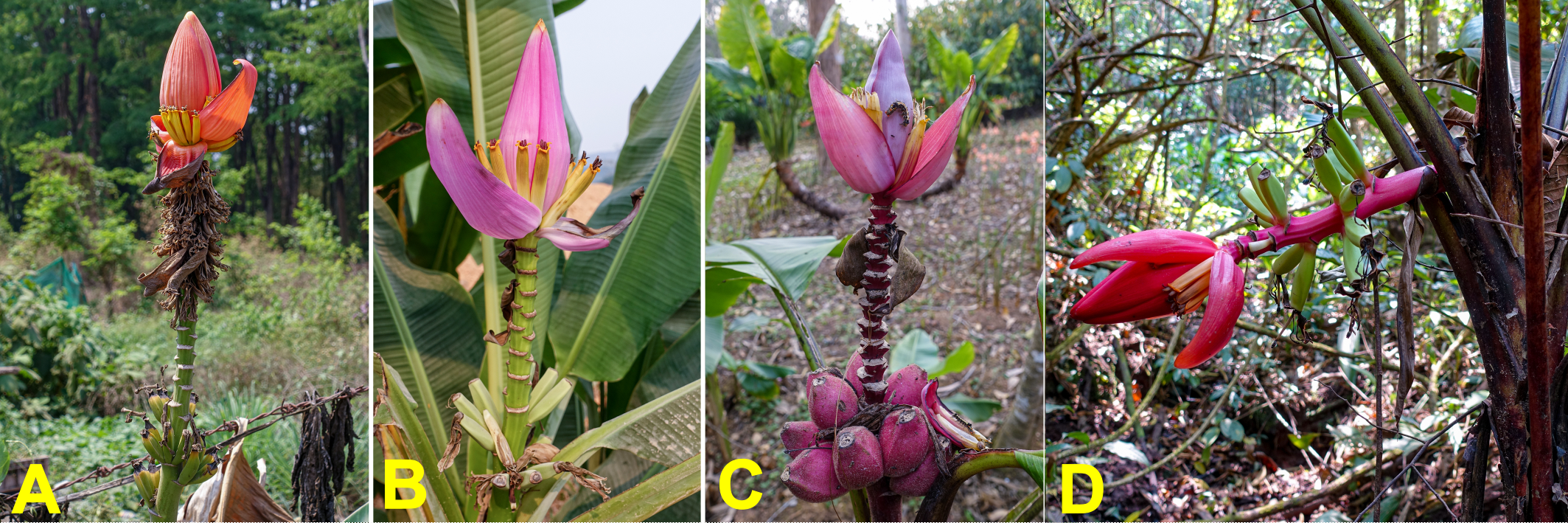

Supplement: S7 Fig — (A) Musa rubra (= ex Musa laterita), (B) Musa ornata, (C) Musa velutina, (D) Musa rubinea (TIF) [file pone.0307592.s009.tif]

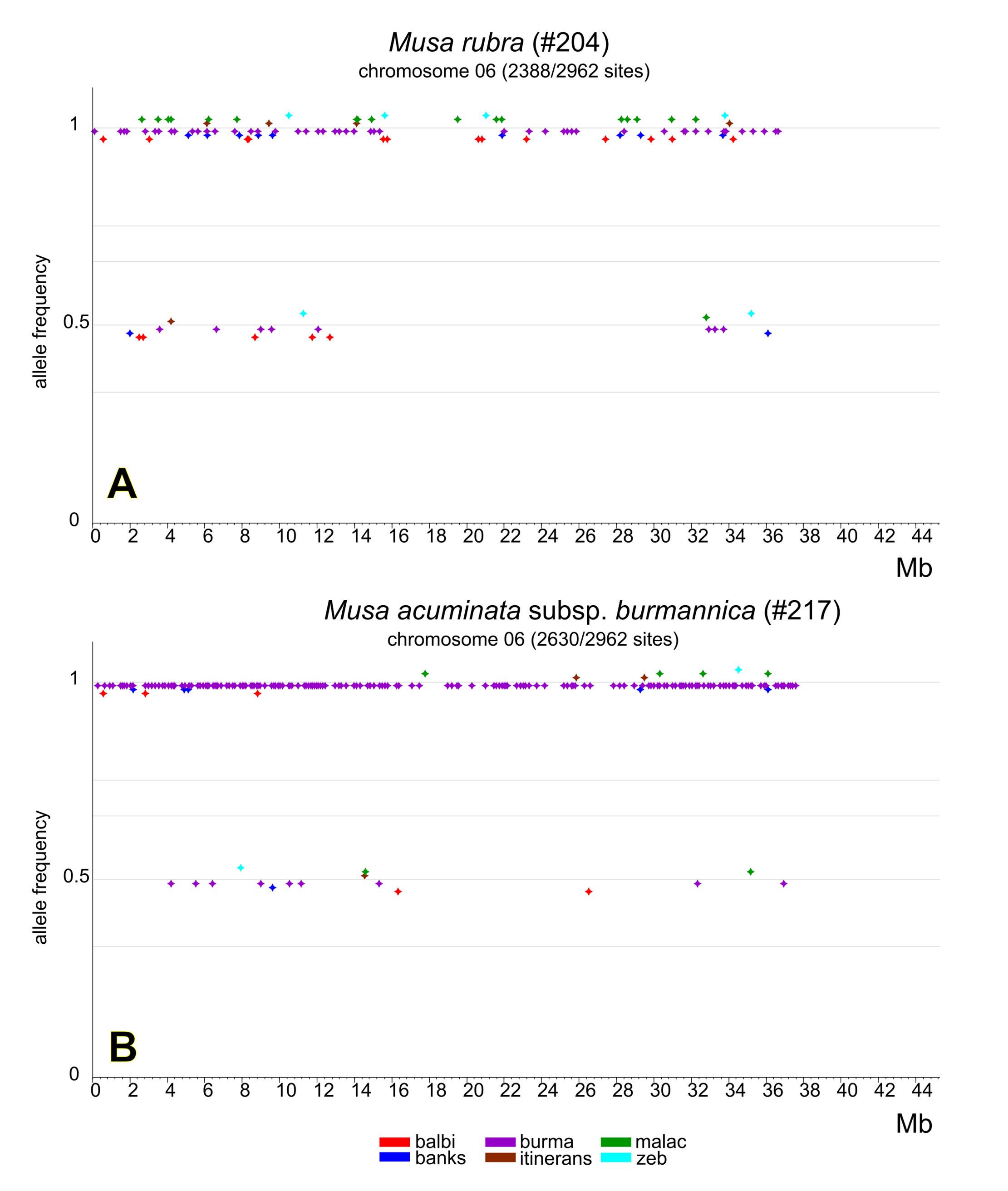

Supplement: S8 Fig — Allelic ratios (0,5 for heterozygotes, 1 for homozygotes) along chromosomes (e.g., chromosome 6) at specific primary pool loci: (A) the collected specimen of M. rubra (#204) and (B) a specimen of M. acuminata subsp. burmannica (#217), for comparison. Colours of primary pools according to the legend. (TIF) [file pone.0307592.s010.tif]
